# Antiviral treatment for treatment-naïve chronic hepatitis B: systematic review and network meta-analysis of randomized controlled trials

**DOI:** 10.1186/s13643-019-1126-1

**Published:** 2019-08-19

**Authors:** William W. L. Wong, Petros Pechivanoglou, Josephine Wong, Joanna M. Bielecki, Alex Haines, Aysegul Erman, Yasmin Saeed, Arcturus Phoon, Mina Tadrous, Mona Younis, Noha Z. Rayad, Valeria Rac, Harry L. A. Janssen, Murray D. Krahn

**Affiliations:** 1 Toronto Health Economics and Technology Assessment Collaborative (THETA), University of Toronto and University Health Network, Toronto, ON Canada; 2 University of Waterloo, School of Pharmacy, Waterloo, ON Canada; 3 University of Toronto, Leslie Dan Faculty of Pharmacy, Toronto, ON Canada; 4 The Hospital for Sick Children, Toronto, ON Canada; 5 The Ontario Drug Policy Research Network, St. Michael’s Hospital, Toronto, ON Canada; 6 BioPharma Services Inc, Toronto, ON Canada; 7 Toronto Centre for Liver Disease, University Health Network, Toronto, ON Canada; 8 Ted Rogers Centre for Heart Research at Peter Munk Cardiac Centre, Toronto General Hospital Research Institute (TGHRI), University Health Network (UHN), Toronto, Canada; 9 Institute of Health Policy, Management and Evaluation (IHPME), Dalla Lana School of Public Health, University of Toronto, Toronto, Canada; 10 Diabetes Action Canada, CIHR SPOR Network, Toronto, Canada

**Keywords:** Chronic hepatitis B, Treatment-naïve, Systematic review, Network meta-analysis

## Abstract

**Background:**

Chronic hepatitis B (CHB) infection poses a significant burden to public health worldwide. Most cases are clinically silent until late in the disease course. The main goal of current therapy is to improve survival and quality of life by preventing disease progression to cirrhosis and liver failure, and consequently hepatocellular carcinoma development. The objective of this review is to provide a contemporary and comprehensive evaluation of the effectiveness of treatment options.

**Methods:**

We performed a systematic review of peer-reviewed literature for randomized controlled trials involving treatment-naïve CHB adult population who received antiviral therapy. The endpoints were virologic response (VR), normalization of alanine aminotransferase (ALT norm), HBeAg loss, HBeAg seroconversion, and HBsAg loss for the HBeAg-positive population; and VR and ALT norm for the HBeAg-negative population. Network meta-analysis (NMA) was performed to synthesize evidence on the efficacy of treatment.

**Results:**

Forty-two publications were selected. Twenty-three evaluated HBeAg-positive population, 13 evaluated HBeAg-negative population, and six evaluated both. We applied NMA to the efficacy outcomes of the two populations separately. Treatment strategies were ranked by the probability of achieving outcomes, and pairwise comparisons calculated from NMA were reported in odds ratios (OR). For HBeAg-positive population, tenofovir disoproxil fumarate (TDF) and tenofovir alafenamide (TAF) were the best for VR; OR vs adefovir = 14.29, 95% CI 7.69–25 and 12.5, 95% CI 4.35–33.33 respectively. TAF was the best for achieving ALT norm (OR vs placebo = 12.5, 95% CI 4.55–33.33), HBeAg loss, and seroconversion (OR vs entecavir/TDF combination = 3.03, 95% CI 1.04–8.84 and 3.33, 95% CI 1.16–10 respectively). In the HBeAg-negative population, TDF and TAF were the best for VR (OR vs adefovir = 9.79, 95% CI 2.38–42.7 and 11.71, 95% CI 1.03–150.48 respectively). Telbivudine and TAF were the best for ALT norm. Certain nucleos(t)ide combinations also had high probability of achieving positive outcomes.

**Conclusions:**

Our results are consonant with current clinical guidelines and other evidence reviews. For both HBeAg-positive and HBeAg-negative populations, TDF and TAF are the most effective agents for virologic suppression, and TAF is effective across all outcomes.

**Electronic supplementary material:**

The online version of this article (10.1186/s13643-019-1126-1) contains supplementary material, which is available to authorized users.

## Background

The growing burden of chronic hepatitis B (CHB) infection poses a significant public health concern worldwide [1]. The main goal of CHB therapy is to improve survival and quality of life by preventing disease progression to cirrhosis and liver failure, and consequently hepatocellular carcinoma development. Additional goals of antiviral therapy are to prevent mother-to-child transmission, hepatitis B reactivation, and the development of hepatitis B virus-associated extrahepatic manifestations [2].

Interferon-alfa (IFN) was the first drug approved by the Food and Drug Administration (FDA) for the treatment of CHB in 1992. In 1998, lamivudine (LAM) was available as the first oral therapy for CHB [3]. However, LAM’s effectiveness has been limited because of the development of antiviral resistance [4]. Over the years, other drugs have become available including pegylated interferon-alfa (PEG-IFN), adefovir (ADV), entecavir (ETV), telbivudine (TBV), tenofovir disoproxil fumarate (TDF), and recently tenofovir alafenamide (TAF) [3, 5]. Among these drugs, in particular, IFN and PEG-IFN can produce long-term immune control without the need for long-term treatment in a small proportion of patients. For other treatments, lifelong administration may be required. Initiating therapy with these medications involves consideration of drug-specific trade-offs such as high and potentially lifelong medication costs, limited adherence, potential side effects, and the risk of antiviral resistance.

ETV, TDF, and TAF were approved for the treatment of CHB by FDA in 2005, 2008, and 2016 respectively [6]. They appeared to have a higher genetic barrier to resistance than the other nucleos(t)ides [4, 7]. Patients have also expressed the need for treatments with higher cure rates, higher barriers to resistance, better side effect profiles, and better coverage by drug plans [8].

In 2010, we conducted a systematic review and Bayesian meta-analysis to evaluate the relative efficacy of the first 12 months of CHB antiviral treatments [9]. We concluded that TDF and ETV were the most potent oral antiviral agents for hepatitis B e antigen (HBeAg)-positive patients, and TDF the most effective for HBeAg-negative patients. Nearly 10 years later, there are more data on long-term treatment and follow-up. Virological breakthrough has been observed with long-term therapy which may or may not be related to the emergence of genotypic resistance [10, 11]. New adverse events have been reported notably drug effects on renal function and bone metabolism [12]. There were also new clinical trial data for the recently approved drug, TAF, and for new drug combinations.

The objective of this review was to provide a comprehensive and contemporary look at the effectiveness of CHB antiviral treatments. Our focus was on treatment-naïve adult patients diagnosed with HBeAg-positive or HBeAg-negative CHB infection without co-infections and without decompensated cirrhosis, hepatocellular carcinoma, and liver transplantation. Patients were not further stratified by their baseline hepatitis B virus (HBV) DNA level, serum alanine aminotransferase (ALT) level, or HBV genotype even though these are recognized predictors of response to certain therapies. This was partly due to sample size considerations and partly because the review was intended to be a comprehensive overview.

## Methods

We conducted a thorough exhaustive search of available literature and identified the evidence from randomized controlled trials (RCTs) investigating the comparative effectiveness among the CHB treatments (PEG-IFN, ADV, LAM, ETV, TBV, TDF, TAF as monotherapy or combination therapy) on treatment-naïve adult populations through a systematic review. We synthesized both direct and indirect evidence on efficacy for all possible comparisons using network meta-analysis (NMA).

The protocol for this systematic review was an extension of our earlier systematic review carried out under the sponsorship of the Ontario Drug Policy Research Network (ODPRN) and reported in 2015 [13].

### Endpoint definitions

Since the eradication of HBV is not really achievable with the currently available treatments, treatment success is usually measured by surrogate endpoints using biomarkers which correlate with improvement in long-term clinical outcome. We included five widely accepted efficacy endpoints for the HBeAg-positive population: virologic response (VR), normalization of alanine aminotransferase level (ALT norm), HBeAg loss, HBeAg seroconversion, and hepatitis B surface antigen (HBsAg) loss; and two efficacy endpoints for the HBeAg-negative population: VR and ALT norm. HBsAg loss was originally included as one of the three efficacy endpoints for the HBeAg-negative population. It was subsequently excluded because the data collected were scarce and insufficient for the performance of network meta-analysis.

The primary objective of current treatment strategies is the induction of long-term suppression of HBV DNA levels [2]. Virologic response (VR) in our study was defined as the attainment of undetectable HBV DNA levels as determined by the polymerase chain reaction (PCR) test for the particular study. Threshold values for undetectable HBV DNA levels according to the method of techniques used for measurement were documented, as they could be a source of heterogeneity. Only studies where the threshold of detection was ≤ 200 IU/ml (1000 copies/ml) were used in the analysis.

ALT normalization, which is achieved in most patients with long-term suppression of HBV replication, is an additional important endpoint [2]. ALT normalization in our review was defined as the reduction of ALT levels to below the upper limit of normal for that study.

The induction of HBeAg loss, with or without anti-HBe seroconversion, in HBeAg-positive CHB patients is a valuable endpoint, as it often represents a partial immune control of the CHB infection [2]. In our study, HBeAg loss was defined as achieving an undetectable level, using the threshold of detection used in each corresponding study; and HBeAg seroconversion was defined as undetectable HBeAg with the presence of anti-HBeAg.

The optimal endpoint of HBsAg loss, with or without anti-HBs seroconversion, indicates profound suppression of HBV replication and viral protein expression. HBsAg loss was defined as achieving an undetectable level, using the threshold of detection used in each corresponding study.

### Systematic review procedures

#### Literature search strategy

This systematic review was designed in accordance with the Preferred Reporting Items for Systematic reviews and Meta-analyses (PRISMA statement) [14] and the protocol followed the PRISMA-P statement [15]. The literature search was performed by an information specialist using a peer-reviewed search strategy. Published literature was identified by searching the following bibliographic databases: Ovid MEDLINE (1946–), Ovid MEDLINE In-Process; Ovid EMBASE (1974–); Cochrane Database of Systematic Reviews (CDSR), the Cochrane Central Register of Controlled Trials; and Web of Science: Science Citation Index Expanded (SCI-EXPANDED) (1900–) and Conference Proceedings Citation Index-Science (CPCI-S) (1990–). Our search started from the date of inception of each database until June 2017. Search terms included controlled vocabulary (MeSH) and text-words in the following three concept areas: chronic hepatitis B (CHB), antiviral agents (pegylated interferon, lamivudine, adefovir, entecavir, telbivudine, tenofovir disoproxil fumarate, tenofovir alafenamide), and the following published and validated filter was applied: randomized control trials (RCTs) [16]. The search was limited to the English language. A detailed search strategy for MEDLINE (Ovid) and final search results are provided in Additional file 1.

#### Study selection criteria

This systematic review included RCTs that compared at least two CHB antiviral treatments or one treatment with placebo/no treatment in adult patients diagnosed with CHB. Studies were selected for inclusion in the systematic review based on the selection criteria presented in Table 1.

**Table 1:** Inclusion and exclusion criteria for the selection of randomized controlled trials

Inclusion criteria	
• Adult patients (≥ 18 years of age) diagnosed with HBeAg-positive and/or HBeAg-negative chronic hepatitis B infection	
• Treatment of interest: adefovir (ADV), entecavir (ETV), lamivudine (LAM), pegylated interferon (PEG-IFN), telbivudine (TBV), tenofovir alafenamide (TAF) , tenofovir disoproxil fumarate (TDF); as monotherapy or as combination therapy	
• Comparators: treatment of interest versus placebo (PLA) or Treatment of interest versus different Treatment of interest	
• Outcomes (Efficacy):	
1. Virologic response (VR)	
2. Alanine aminotransferase normalization (ALT norm)	
3. HBeAg loss	
4. HBeAg seroconversion	
5. HBsAg loss	
• Study design: published, randomized, controlled interventional studies	
Exclusion criteria	
• Studies that did not meet the aforementioned selection criteria, uncontrolled non-randomized studies, qualitative studies, observational studies, duplicate publications, conference abstracts, narrative reviews, and editorials were excluded	
• Studies not conducted in English	
• Studies on special populations: coinfections with HIV or other forms of hepatitis, decompensated cirrhosis, hepatocellular carcinoma, liver transplantation, and pregnancy	
• Treatment duration shorter than 48 weeks (except for Interferon therapy)	
• Treatment experienced population > 50%	

*HBeAg* hepatitis B e-antigen, *HBsAg* hepatitis B s-antigen

#### Study screening and quality assessment

Two reviewers (AE and YS) independently screened the titles and abstracts of the identified studies to determine if they met the inclusion criteria, using a hierarchical screening method adapted from PRISMA. Subsequently, the full text of the eligible studies was assessed by the same reviewers independently. Disagreements between the two reviewers (AE and YS) were resolved by discussion. Clinical experts were consulted and existing systematic reviews and meta-analyses were reviewed to identify any relevant studies that were missed. Each included RCT was assessed using the Cochrane risk of bias tool [17] on six validity domains: selection (sequence generation, allocation concealment), performance (blinding of participants and personnel), detection (blinding of outcome assessment), attrition, reporting, and other sources of bias.

#### Data extraction

Using a standard spreadsheet, the following data were extracted: (1) study design, (2) sample size, (3) patient characteristics, (4) treatment doses, (5) treatment and/or follow-up duration, and (6) efficacy outcome measures.

### Data analysis methods

Network meta-analysis (NMA) methods were used to synthesize the evidence from the RCTs on relative effectiveness across the treatment options. Binomial likelihood functions were assumed for the binary events of interest, and a random effects model was used for all the outcomes. The NMA was conducted within a Bayesian framework using JAGS v.4.3.0 [18] and the R package “gemtc” [19]. Under this framework, the distribution of each parameter of interest (posterior distribution) was estimated through a Markov chain Monte Carlo simulation method. We ran three Markov chains and performed 70,000 simulations for each chain for each outcome, and we excluded the first 20,000 simulations to ensure that we selected only converged values. We further assessed the convergence for all models through the Gelman and Rubin diagnostic [20]. Vague priors were assumed throughout the model. Normal priors were assumed for treatment effectiveness parameters with a mean zero and a variance of 10,000, while a uniform prior U(0,2) was assumed for the standard deviation of the heterogeneity parameter. Median estimates for the parameters of interest together with their 95% credible intervals (95% CrI) were constructed from the posterior distributions of the MCMC simulations. In addition, the probability of each treatment to be the best, second best, third best, etc. was presented for all treatment options using rankograms.

An important assumption underlying NMA is that of consistency between the direct and indirect evidence of relative efficacy. Inconsistency check evaluates the validity of a network meta-analysis by assessing the compatibility of direct and indirect evidence. If the results from direct evidence conflict with the results from indirect evidence then this highlights a problem of inconsistency in the network. In this case, one could argue that inconsistency in the network, that is the direct and indirect evidence, may not be compatible. Any presence of inconsistency in closed loops was assessed through the use of the node-split method [21]. The difference in effect size between the direct and indirect evidence was estimated and tested to see if the difference was significantly different from zero. Any *p* values below 0.05 would indicate that the difference in effect size between direct and indirect evidence was statistically significant from zero and therefore inconsistency existed in the model. In the case where inconsistency was identified, the studies involved in the inconsistent loops of the network were reviewed to detect any clinical heterogeneity that might have caused the inconsistency.

Sensitivity analysis was performed to assess the impact of assumptions related to the type of model (fixed vs random) on the estimates of relative efficacy (Additional file 6).

NMA was conducted on five efficacy outcomes in the HBeAg-positive population: HBV DNA suppression (VR), ALT norm, HBeAg loss, HBeAg seroconversion, and HBsAg loss; and on two efficacy outcomes in the HBeAg-negative population: VR and ALT norm. Based on the NMA, we estimated the probability for each treatment strategy to achieve each outcome and the odds ratios (OR) of pairwise comparisons between treatment options. In addition, the probability of each treatment to be the best, second best, third best, etc. was presented for all treatment options using rankograms.

In the main NMA, we included studies with combination strategies as long as they were given to naïve CHB patients.

## Results

### Selection of randomized control trials

A total of 6319 studies were identified from the original literature (Additional file 1). After screening the titles and abstracts, 1040 potentially relevant publications were retrieved for full text review (kappa = 0.733; good agreement). After full text review, 42 publications were selected for inclusion (kappa = 0.94; very good agreement). Additional file 2 is a summary of the risk of bias assessment of the included studies and Additional file 3 describes the included study characteristics and the abbreviations for treatment strategies used in NMA. These 42 publications were for mono-infected treatment-naïve adult patients. Twenty-three publications evaluated HBeAg-positive population only [22–44] and 13 publications evaluated HBeAg-negative population only [45–57]. Six publications assessed both populations [58–63] and were included in both networks as they reported the outcomes separately for each population. Table 2 summarizes the included studies by interventions according to the HBeAg status. The total number of participants in each study ranged from 42 to 921. The overall number of included participants was 12,885. Participant baseline characteristics in each study are summarized in Additional file 4.

**Table 2:** Summary of included studies by treatments according to HBeAg status

Treatments included in the network meta-analysis	Studies (*n*)	DB RCT (*n*)	Patients (*n*)	Publication years
HBeAg-positive patients				
Lamivudine (LAM)	12	9	1925	1998–2015
Adefovir (ADV)	6	3	652	2003–2015
Telbivudine (TBV)	4	3	692	2005–2014
Entecavir (ETV)	9	3	1127	2006–2017
Tenofovir disoproxil fumarate (TDF)	6	4	1065	2008–2017
Tenofovir alafenamide (TAF)	1	1	581	2016
Pegylated interferon (PEG-IFN)	6	2	727	2005–2016
Combination therapies	14	4	1740	2005–2016
Placebo (PLA)	4	3	330	1998–2014
Total	29^a^	15^a^	8839	1998–2017
HBeAg-negative patients				
Lamivudine (LAM)	9	8	1005	1999–2017
Adefovir (ADV)	3	3	401	2003–2015
Telbivudine (TBV)	2	2	242	2009–2014
Entecavir (ETV)	4	3	470	2006–2017
Tenofovir disoproxil fumarate (TDF)	3	3	544	2008–2016
Tenofovir alafenamide (TAF)	1	1	285	2016
Pegylated interferon (PEG-IFN)	6	2	375	2004–2016
Combination therapies	7	2	525	2004–2016
Placebo (PLA)	4	3	180	1999–2007
Total	19^a^	13^a^	4046	1999–2017

*HBeAg* hepatitis B e antigen, *n* number, *DB RCT* double-blind randomized controlled trial

^a^Unique studies

As for the 998 articles excluded, the reasons for exclusion were lack of comparators or control (36%); no outcomes of interest reported (28%); duplicate papers, secondary analyses, modeling studies or abstracts (20%); and studies on special subsets of patients such as co-infections, decompensated cirrhosis, hepatocellular carcinoma, hemodialysis, and transplant (5%).

### Network meta-analyses for RCT: efficacy

Initially, we have included eight monotherapies and 19 combination treatment strategies in the NMA for HBeAg-positive patients; and eight monotherapies and six combination treatment strategies in the NMA for HBeAg-negative patients. However, after conducting the inconsistency analysis, we identified evidence for significant inconsistencies (Additional file 5) in some of the network loops for the efficacy results. After review of the relevant studies for the loops with evidence for inconsistency, we determined that the inconsistencies were likely to have originated from the fact that the included treatment PEG-IFN had a different mechanism of action from the oral nucleos(t)ides. Among these studies, PEG-IFN was used in different dosages and schedules and was combined in different order with oral nucleos(t)ides in combination therapy. On the basis of the clinical heterogeneity, we decided to further exclude studies that included the PEG-IFN treatment.

**Fig. 1:**
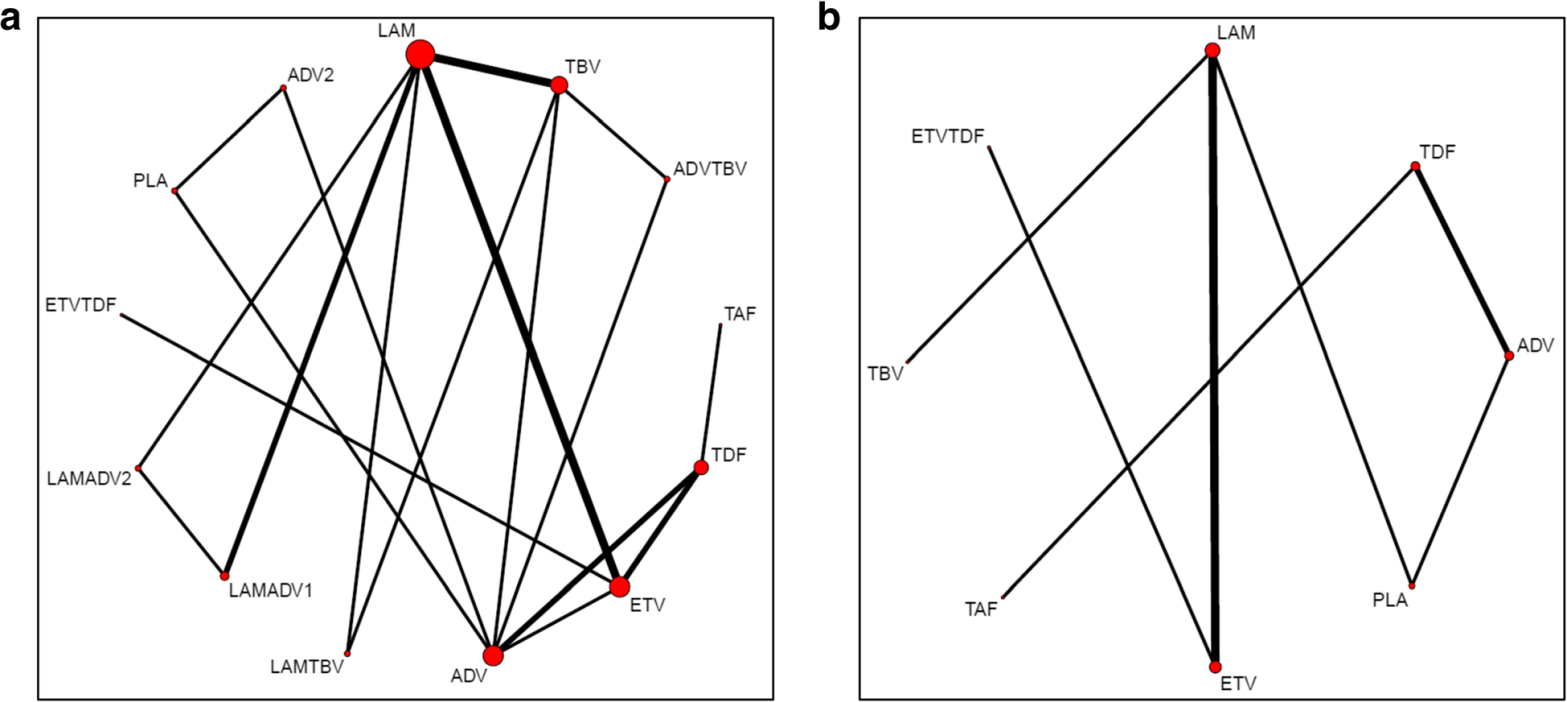
**a** Evidence network for virologic response for HBeAg-positive patients. **b** Evidence network for virologic response for HBeAg-negative patients. *ADV* adefovir 10 mg daily, *ADV2* adefovir 30 mg daily, *ETV* entecavir, *LAM* lamivudine, *TBV* telbivudine, *PLA* placebo, *TAF* tenofovir alafenamide, *TDF* tenofovir disoproxil fumarate; ADVTBV, ETVTDF, LAMADV1, LAMADV2, LAMTBV, and ETVTDF code for different combinations of antiviral agents with full details in Additional file 3

**Fig. 2:**
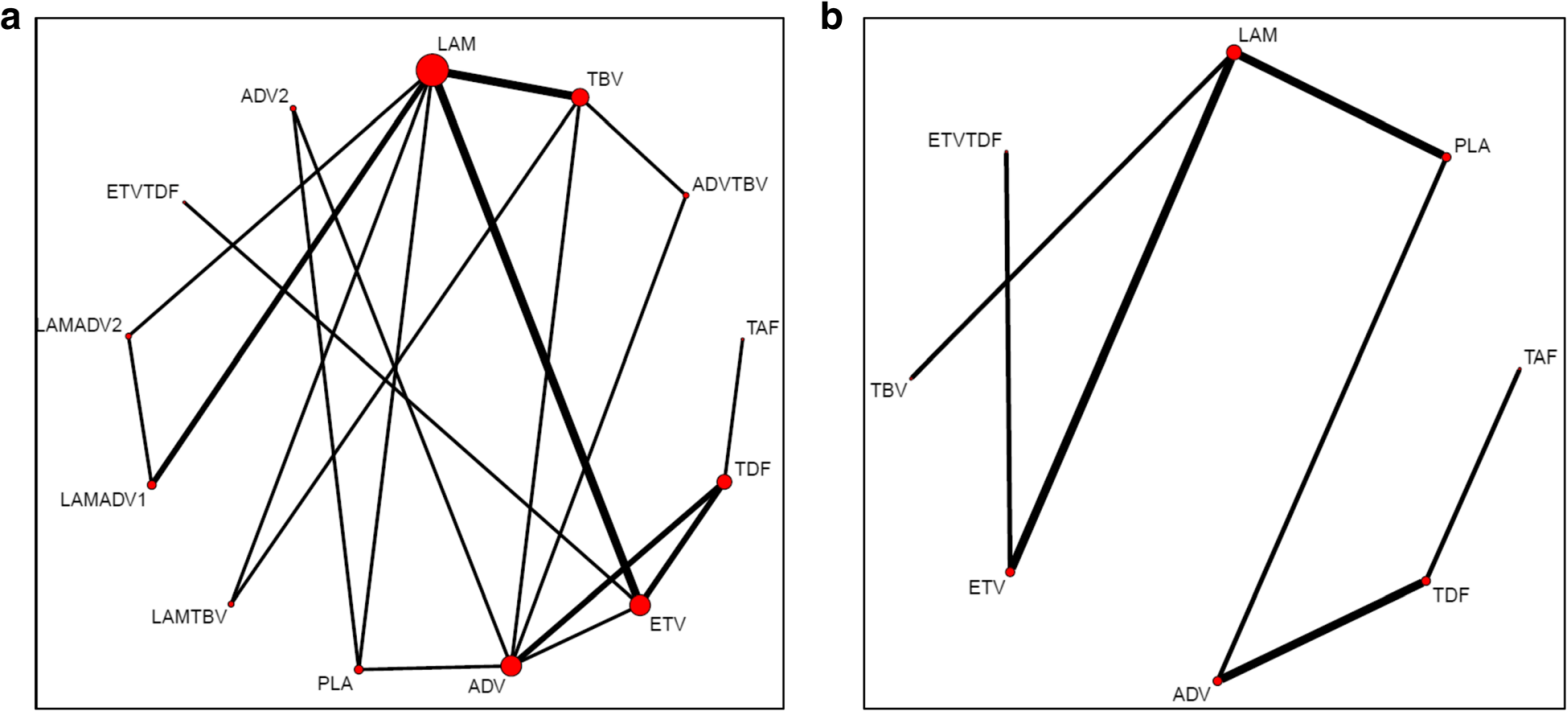
**a** Evidence network for ALT normalization for HBeAg-positive patients. **b** Evidence network for ALT normalization for HBeAg-negative patients. *ADV* adefovir 10 mg daily, *ADV2* adefovir 30 mg daily, *ETV* entecavir, *LAM* lamivudine, *TBV* telbivudine, *PLA* placebo, *TAF* tenofovir alafenamide, *TDF* tenofovir disoproxil fumarate; ADVTBV, ETVTDF, LAMADV1, LAMADV2, LAMTBV, and ETVTDF code for different combinations of antiviral agents with full details in Additional file 3

**Fig. 3:**
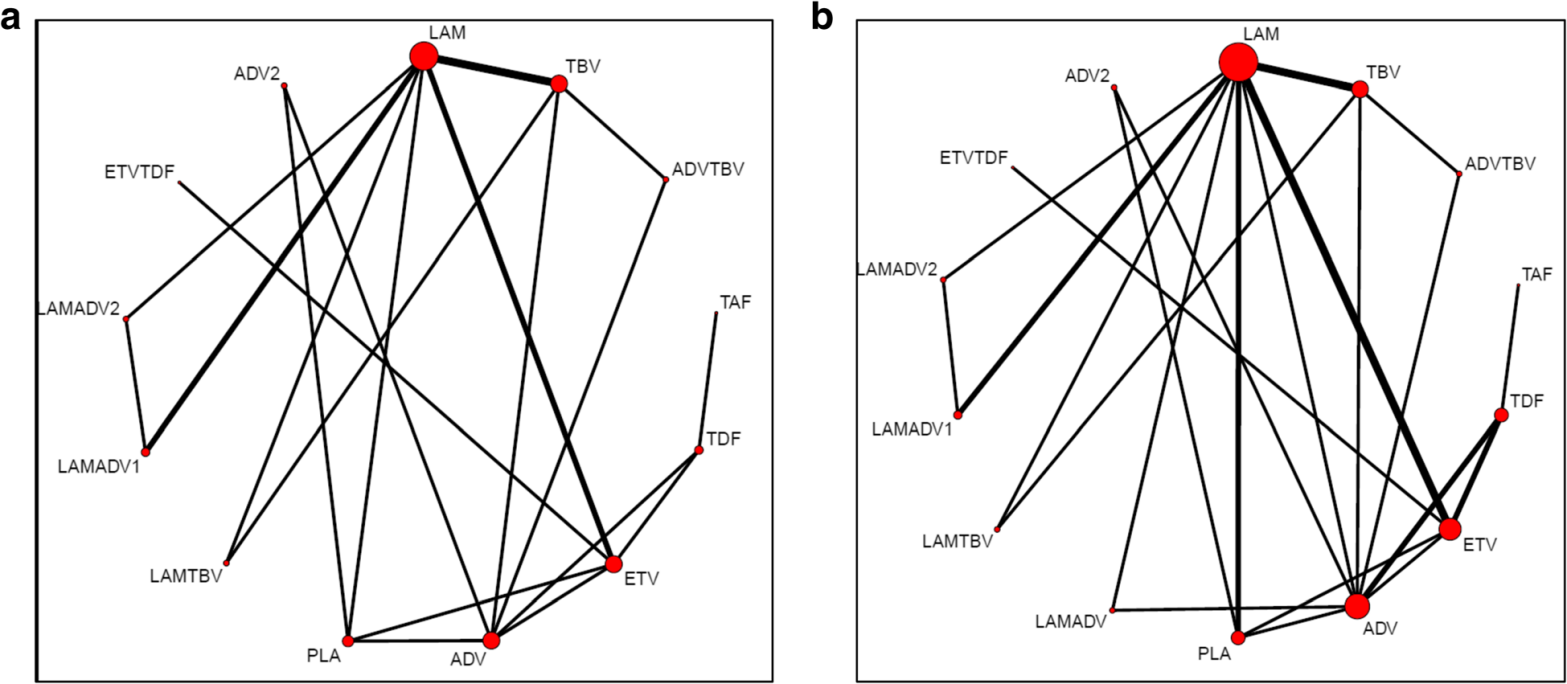
**a** Evidence network for HBeAg loss for HBeAg-positive patients. **b** Evidence network for HBeAg seroconversion for HBeAg-positive patients. *ADV* adefovir 10 mg daily, *ADV2* adefovir 30 mg daily, *ETV* entecavir, *LAM* lamivudine, *TBV* telbivudine, *PLA* placebo, *TAF* tenofovir alafenamide, *TDF* tenofovir disoproxil fumarate; ADVTBV, ETVTDF, LAMADV, LAMADV1, LAMADV2, and LAMTBV code for different combinations of antiviral agents with full details in Additional file 3

**Fig. 4:**
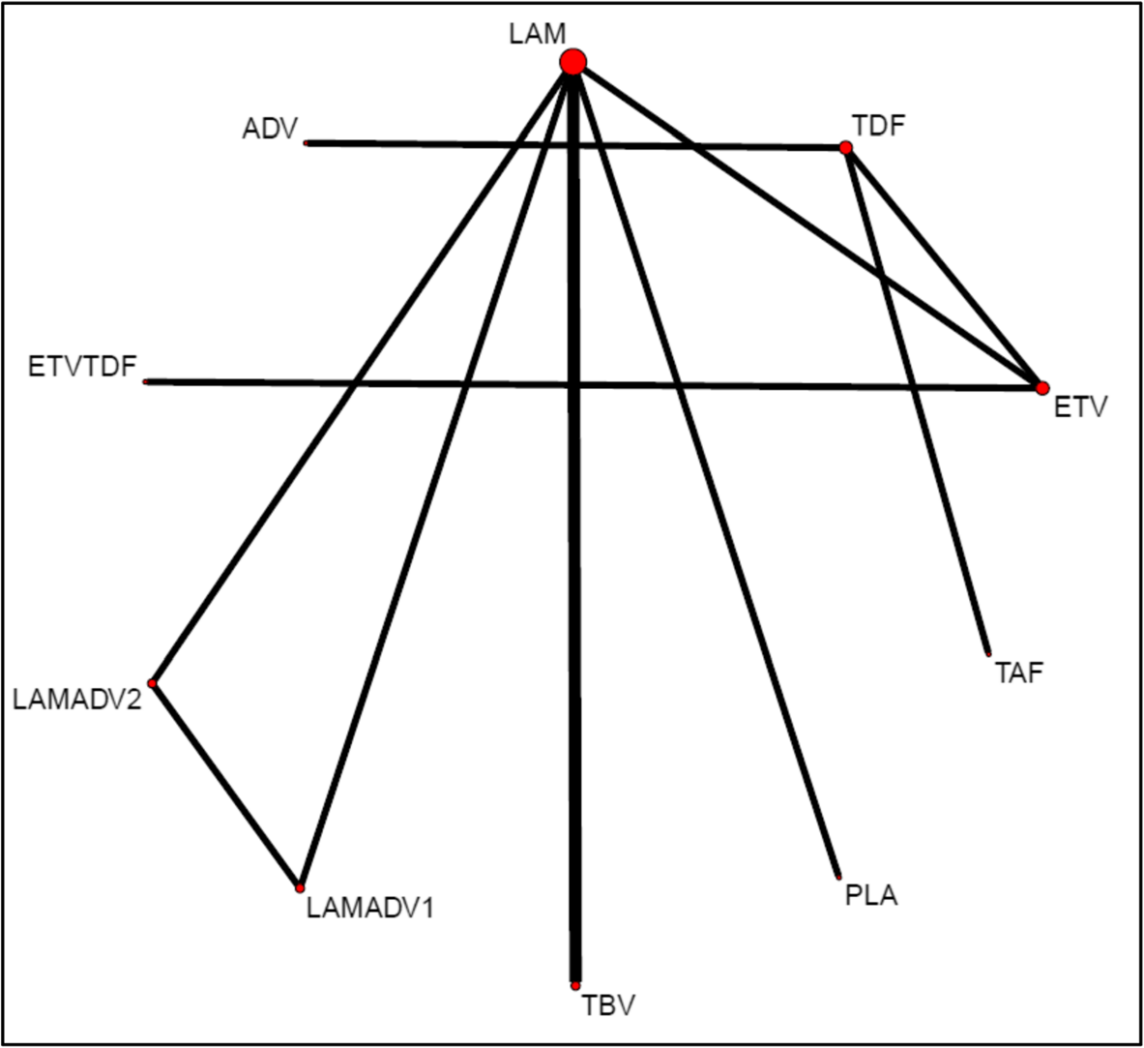
Evidence network for HBsAg loss for HBeAg-positive patients. *ADV* adefovir 10 mg daily, *ETV* entecavir, *LAM* lamivudine, *TBV* telbivudine, *PLA* placebo, *TAF* tenofovir alafenamide, *TDF* tenofovir disoproxil fumarate; ETVTDF, LAMADV1, and LAMADV2 code for different combinations of antiviral agents with full details in Additional file 3

**Fig. 5:**
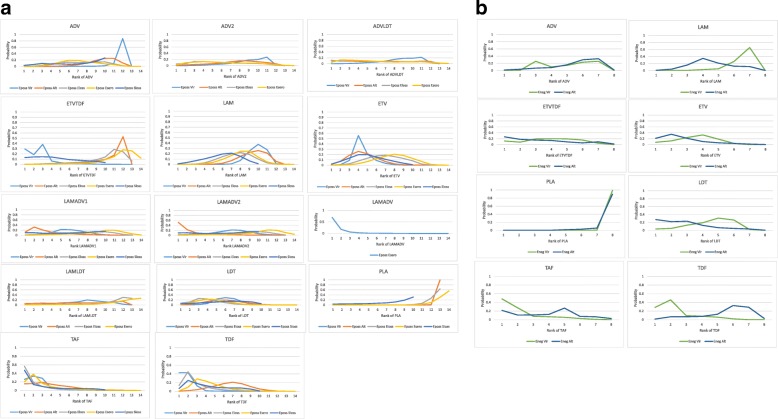
**a** Cumulative rankograms generated by network meta-analysis of treatments for HBeAg-positive population. **b** Cumulative rankograms generated by network meta-analysis of treatments for HBeAg-negative population. *HBeAg* hepatitis B e-antigen, *VR* virologic response, *ALT norm* normalization of serum alanine aminotransferase, *HBeAg loss* hepatitis B e-antigen loss, *HBeAg sero* hepatitis B e-antigen seroconversion, *HBsAg loss* hepatitis B surface antigen loss, *ADV* adefovir 10 mg daily, *ADV2* adefovir 30 mg daily, *ETV* entecavir, *LAM* lamivudine, *TBV* telbivudine, *PLA* placebo, *TAF* tenofovir alafenamide, *TDF* tenofovir disoproxil fumarate; ADVTBV, ETVTDF, LAMADV, LAMADV1, LAMADV2, and LAMTBV code for different combinations of antiviral agents with full details in Additional file 3

In the final NMA, we included seven monotherapies and six combination treatment strategies in the NMA for HBeAg-positive patients; and seven monotherapies and one combination treatment strategy in the NMA for HBeAg-negative patients. The evidence networks are presented in Fig. 1a, b for virologic response, Fig. 2a, b for ALT normalization, Fig. 3a, b for HBeAg loss and seroconversion respectively, and Fig. 4 for HBsAg loss. In these evidence networks, the size of the circles corresponds to the number of patients exposed to a treatment and the thickness of the connecting lines corresponds to the number of studies comparing the treatments directly. The relative efficacy in terms of odds ratios and 95% credible intervals of pairwise comparisons for each outcome are presented in Tables 3 and 4. The probability of each treatment being the 1st, 2nd, 3rd, etc. are provided in the form of rankograms in Fig. 5a, b for the e-positive and e-negative populations respectively. We also compared the variances across all the node split models for each outcome against the consistent model. There were no significant fluctuations of the variance and its confidence interval across all models, with large overlap across the confidence intervals. This finding indicated limited evidence of inconsistency.

**Table 3:**
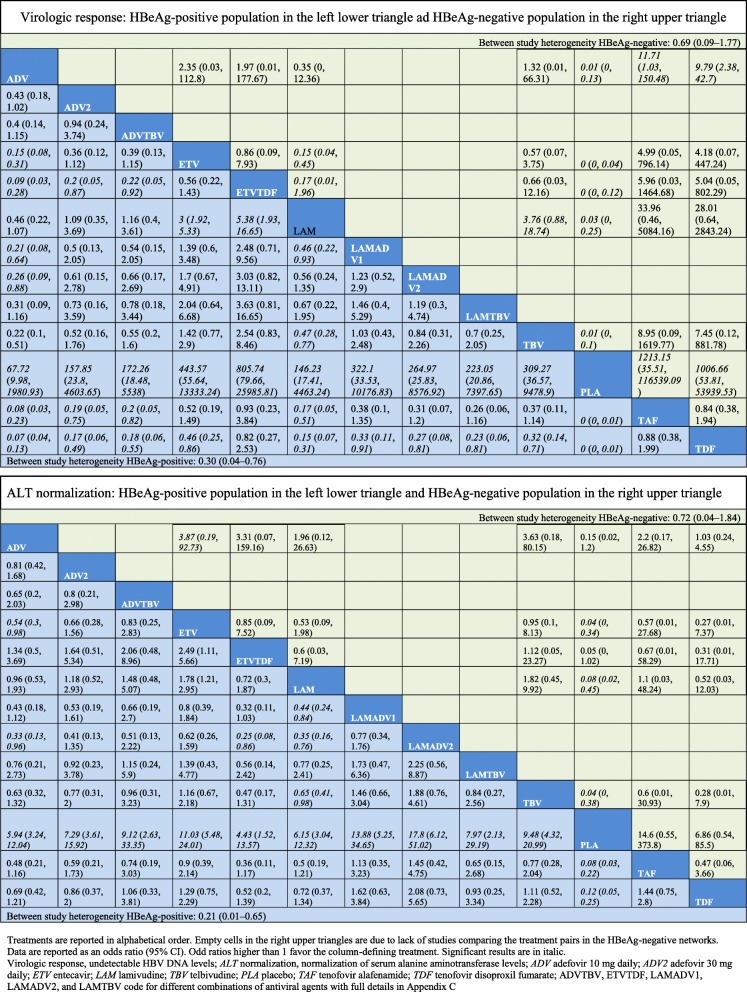
Relative effects on virologic response and ALT normalization of all pairs of interventions in odds ratios (95% credible intervals) as calculated from the network meta-analyses using random effects models

**Table 4:**
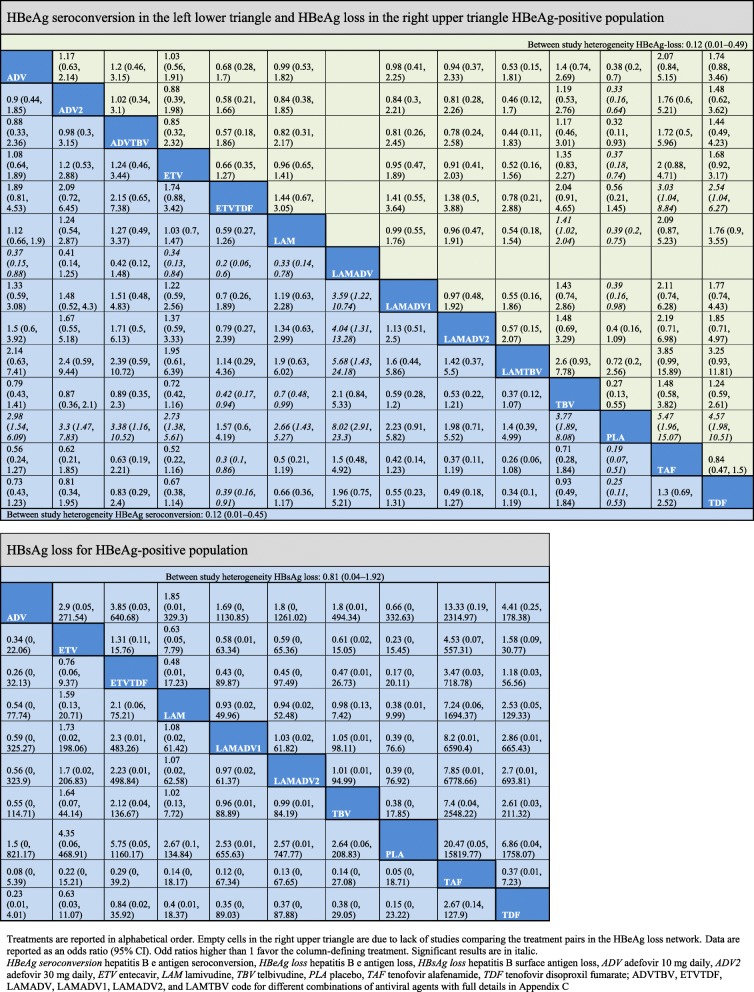
Relative effects on serological responses of all pairs of interventions in odds ratios (95% credible intervals) as calculated from the network meta-analyses using random effects models

#### HBeAg positive patients: viral suppression and ALT normalization

In pairwise comparisons across all drugs (Table 3), TDF had statistically significantly higher odds of achieving viral suppression than the rest except for ETVTDF and TAF, e.g., ETV vs TDF, OR = 0.46, 95% CrI 0.25–0.86; TAF vs TDF, OR = 0.88, 95CrI 0.38–1.99. TDF had a probability of 43% being the best treatment for achieving virologic response, followed by the combination strategy ETVTDF (29%) and TAF (26%) (Fig. 5a). For ALT normalization, pairwise comparisons (Table 3) showed that no one drug was consistently superior. The combination LAMADV2 was significantly better than LAM (OR = 0.35, 95% Crl 0.16–0.76), ADV (OR = 0.33, 95% Crl 0.13–0.96), and ETVTDF (OR = 0.25, 95% CrI 0.08–0.86). LAMADV2 had a probability of 52% being the best treatment followed by TAF (16%) (Fig. 5a).

#### HBeAg-positive patients: HBeAg loss, HBeAg seroconversion, and HBsAg loss

For HBeAg seroconversion, pairwise comparison (Table 4) showed that LAMADV was significantly better than LAMADV1 (OR = 3.59, 95% CrI 1.22–10.74), LAMADV2 (OR = 4.04, 95% CrI 1.31–13.28), and LAMTBV (OR = 5.68, 95% CrI 1.43–24.18); and TAF was significantly better than ETVTDF (OR = 0.3, 95% CrI 0.1–0.86). LAMADV had the highest probability (69%), followed by TAF (21%) (Fig. 5a). For HBeAg loss (Table 4), TAF was significantly better than ETVTDF (OR = 3.03, 95% CrI 1.04–8.84) and had the highest probability of achieving outcome (57%) (Fig. 5a). With respect to HBsAg loss, no one drug significantly outperformed the others (Table 4).

#### HBeAg negative patients: viral suppression and ALT normalization

For virologic response, TAF and TDF had the highest probabilities of achieving viral suppression (48% and 28% respectively) (Fig. 5b). However, pairwise comparisons across drugs (Table 3) did not show consistent superiority except comparing with ADV. For ALT normalization, TBV had the highest probability (27%) of achieving this outcome (Fig. 5b). This was followed by ETVTDF (26%) and TAF (22%). However, pairwise comparisons across drugs (Table 3) did not show consistent superiority.

## Discussion

We used network meta-analysis to integrate all available randomized trial evidence for the treatment of CHB by conducting direct and indirect comparisons among multiple treatment strategies. For each treatment within the Bayesian framework, we estimated the posterior probability of achieving a specific outcome and presented the results in the form of rankogram. However, we have to interpret this probability ranking with caution. The choice of the most desirable treatment should not be based solely on the ranking, but should take the relative effect into account [64].

Treatment recommendations depend not only on effectiveness but also on many other factors such as adverse events, potential for viral resistance, cost, patients’ preference and values, and availability in the care setting. In addition, the choice has to be made across five relevant outcomes for HBeAg-positive patients and two outcomes for HBeAg-negative patients. A treatment that is best in one outcome may not fare well in another outcome.

Across all outcomes and in both HBeAg-positive and HBeAg-negative populations, TAF emerged as the treatment with the most consistent performance (Fig. 5a, b). TAF was approved by FDA in 2016 for the treatment of CHB. It is a prodrug of tenofovir and has a better safety profile with respect to renal function and bone mineral density compared to TDF [65–68]. It is rapidly converted to the active metabolite intracellularly. With reduced systemic exposure to the active metabolite, the off-target kidney and bone exposure are thereby reduced. It has the same resistance profile as TDF [69]. However, TAF is costly (estimated US$15,570.90 per patient annually at US average wholesale price of $42.66/25 mg tablet) [70], though one published study has suggested that TAF may nonetheless be cost effective [71].

Prior to 2017, TDF and ETV were the recommended oral drugs for CHB in the international guidelines because they have high effectiveness and a high barrier to resistance [72, 73]. With TDF, renal complications (nephropathy and Fanconi syndrome) and osteomalacia have been reported [74], but no resistance has been detected to date [7]. ETV has been associated with lactic acidosis and a low but slowly emerging resistance pattern [74, 75].

We found significant inconsistencies when studies with PEG-INF were included in the NMA with oral nucleos(t)ides. Among these studies, PEG-IFN was used in different dosages; the duration of treatment was shorter than the oral nucleos(t)ides, and PEG-INF was combined in different order with oral nucleos(t)ides in different combination therapies. This means that there were effect modifiers in the identified studies that suggested the evidence should not be combined. For the purpose of this NMA, the studies identified were too heterogeneous to combine, which manifested as inconsistency in the network. On the basis of this clinical heterogeneity, we decided to further exclude studies that included the PEG-IFN treatment.

Pegylated interferon-α (PEG-IFN) is a synthetic cytokine and is believed to act on the cell-mediated immunity [76]. PEG-IFN treatment has the benefit of finite treatment duration, a higher rate of HBeAg and HBsAg seroconversion, and no drug resistance [77]. However, it is associated with more adverse events (flu-like symptoms, neutropenia, anemia, thrombocytopenia, depression, neuropathy, and dermatological side effects) than any of the oral drugs [78]. The need for parenteral administration also adds to the low preference and compliance. For all these reasons, PEG-IFN is hardly used in clinical practice nowadays. It has also been shown that extended nucleos(t)ide therapy could result in HBsAg loss (functional cure) rates similar to those reported with PEG-IFN therapy [79]. In recent years, using regression analysis, clinicians are able to identify and select specific patients with positive predictors for sustained response to PEG-IFN and reduce unnecessary exposure for patients who are not likely to respond [80, 81]. Certain combination strategies between a nucleos(t)ide and PEG-IFN have been shown to exhibit synergistic therapeutic effect resulting in greater viral suppression and higher rates of HBeAg loss and HBsAg loss [82, 83].

In this review, we included all eligible RCTs published prior to the end of 2017. Our results are consonant with current clinical guidelines [2, 72, 84] which recommend TAF, TDF, and ETV to be the first-line treatment for CHB, and PEG-IFN for selective patient groups. These recommendations are based on evidence reviews, consensus of expert panels, and ratings on the Grading of Recommendations, Assessment, Development and Evaluation system. Our NMA provides comprehensive comparative evidence for these treatment recommendations.

Our analysis is also broadly consonant with other evidence reviews. In comparison with the NMAs published in 2010 and 2015 [9, 85], this analysis has included a new drug, TAF, and many new combination strategies. There are some differences in ranking. All three NMAs ranked TDF first for virologic suppression for HBeAg-positive patients. For ALT normalization, the two previous NMAs ranked TDF first for HBeAg-positive patients and TBV first for HBeAg-negative patients. We ranked TAF first for HBeAg-positive patients and TBV first for HBeAg-negative patients.

Our study is in agreement with the findings of another two NMAs in that TBV was superior to most other nucleos(t)ides in terms of HBeAg loss and seroconversion [86, 87]. This was until the arrival of TAF which superseded TBV.

Since our previous review in 2010, drug combinations have been investigated increasingly to delay the emergence of resistance, to treat patients with previous treatment failure [88, 89], and most importantly to increase immune control over the virus, as indicated by HBeAg loss and HBsAg loss [38, 44]. However, there are relatively few studies at present, and sample sizes are small.

Our study has a few limitations that merit discussion. Firstly, we included studies that spanned 19 years. The laboratory methods used for quantification of HBV DNA have evolved tremendously in the last decade with detection threshold as low as < 10 IU/ml (approximately 50 copies/ml). Consequently, the definition for virologic response varied widely between older studies and recent studies and ranged from < 20,000 IU/ml (100,000 copies/ml) to < 15 IU/ml (75 copies/ml). Even though we only included in our analysis those outcome data with detection limits of ≤ 200 IU/ml (1000 copies/ml), this is still an important source of variability. Secondly, in order to include as many interventions as possible to inform the network, we did not specify participants’ baseline characteristics in our eligibility criteria. This could be an important source of heterogeneity. However, we tried to reduce baseline variability by excluding studies with decompensated cirrhosis, hepatocellular carcinoma, and liver transplant cohorts. Additional file 4 showed the variation in age, gender, baseline HBV DNA level, and baseline ALT level among the included studies. Studies with more favorable patient selection criteria are expected to produce more favorable results. Viral suppression is highly dependent on the baseline HBVDNA levels and treatment duration. It has been shown that baseline HBV DNA and HBsAg levels are strong predictors of virologic response to different CHB treatments [90–92]. ALT normalization is highly dependent on the inclusion criteria of ALT level and the presence of concomitant disease such as nonalcoholic steatohepatitis. Other baseline variables such as HBV genotypes also have correlation with treatment outcome [93, 94]. This variation in baseline clinical characteristics is a recognized source of heterogeneity in NMA [95, 96]. Thirdly, the small number of studies and patients limited the ability to conduct pairwise comparisons. This is particularly true for combination therapies. These small sample sizes produced wide credible intervals and reduced the number of closed loops in the network, especially for the HBeAg-negative population. This is also true for outcomes with rare events such as HBsAg loss, where the observed wide credible intervals indicate large uncertainty in differences between treatments. Fourthly, for some studies where information comes from fewer studies and more rare events, the estimate of between-study heterogeneity is more heavily dependent on the choice of the (vague) prior. As a consequence, the estimate of uncertainty for some of the relative effectiveness parameters is inflated. The mean estimates or relative effects, however, have remained unchanged. Lastly, given that our review was limited to studies written in English, useful studies in other languages may have been missed.

## Conclusions

The currently available treatment options are either nucleos(t)ide-based or interferon-based or their combinations. For both hepatitis B e antigen-positive and -negative populations, tenofovir disoproxil fumarate and tenofovir alafenamide are the most effective agent for virologic suppression. Our NMA confirmed the low probabilities and overall difficulty of achieving such endpoints as HBeAg loss/seroconversion or HBsAg loss. Even when virologic suppression is achieved, relapse rate is high and long-term treatment is necessary. Research for new treatment strategies and targets is necessary to achieve a functional cure. Currently, many new potential drugs targeting directly at the genomic viral reservoirs (cccDNA and integrated viral DNA) and agents boosting innate and HBV-specific immunity are in the pipelines [97–100]. Until these new therapies arrive, best practice for care of HBV patients should be based on current evidence, as reported here.

## Additional files

Additional file 1: PRISMA flow diagram and full search strategy for Ovid MEDLINE. (PDF 623 kb)

Additional file 2: Cochrane risk of bias assessment results. (PDF 375 kb)

Additional file 3: Characteristics of included randomized controlled trials. (PDF 358 kb)

Additional file 4: Participant baseline characteristics of included randomized controlled trials. (PDF 398 kb)

Additional file 5: Network inconsistency evaluation. (PDF 59 kb)

Additional file 6: Relative effects on outcomes of all pairs of interventions in Odds Ratios (95% credible intervals) as calculated from the network meta-analyses using fixed effects models. (PDF 122 kb)

## Data Availability

The datasets used and/or analyzed in this study are available from the corresponding author on reasonable request.

## Abbreviations

ADV: Adefovir
ALT: Alanine transaminase
CHB: Chronic hepatitis B
ETV: Entecavir
HBeAg: Hepatitis B e antigen
HBsAg: Hepatitis B surface antigen
HBV: Hepatitis B virus
HCC: Hepatocellular carcinoma
LAM: Lamivudine
NMA: Network meta-analysis
PEG-IFN: Pegylated interferon alfa
RCT: Randomized controlled trial
TAF: Tenofovir alafenamide
TBV: Telbivudine
TDF: Tenofovir disoproxil fumarate
