# Supplementary material for: Antiviral treatment for treatment-naïve chronic hepatitis B: systematic review and network meta-analysis of randomized controlled trials
Source: Syst Rev. 2019 Aug 19;8:207. doi: 10.1186/s13643-019-1126-1 (PMC6699129; doi:10.1186/s13643-019-1126-1)
Supplement: Supplementary file 1 — PRISMA flow diagram and full search strategy for Ovid MEDLINE. (PDF 623 kb) [file 13643_2019_1126_MOESM1_ESM.pdf]

Appendix A: PRISMA flow diagram and full search strategy for Ovid MEDLINE

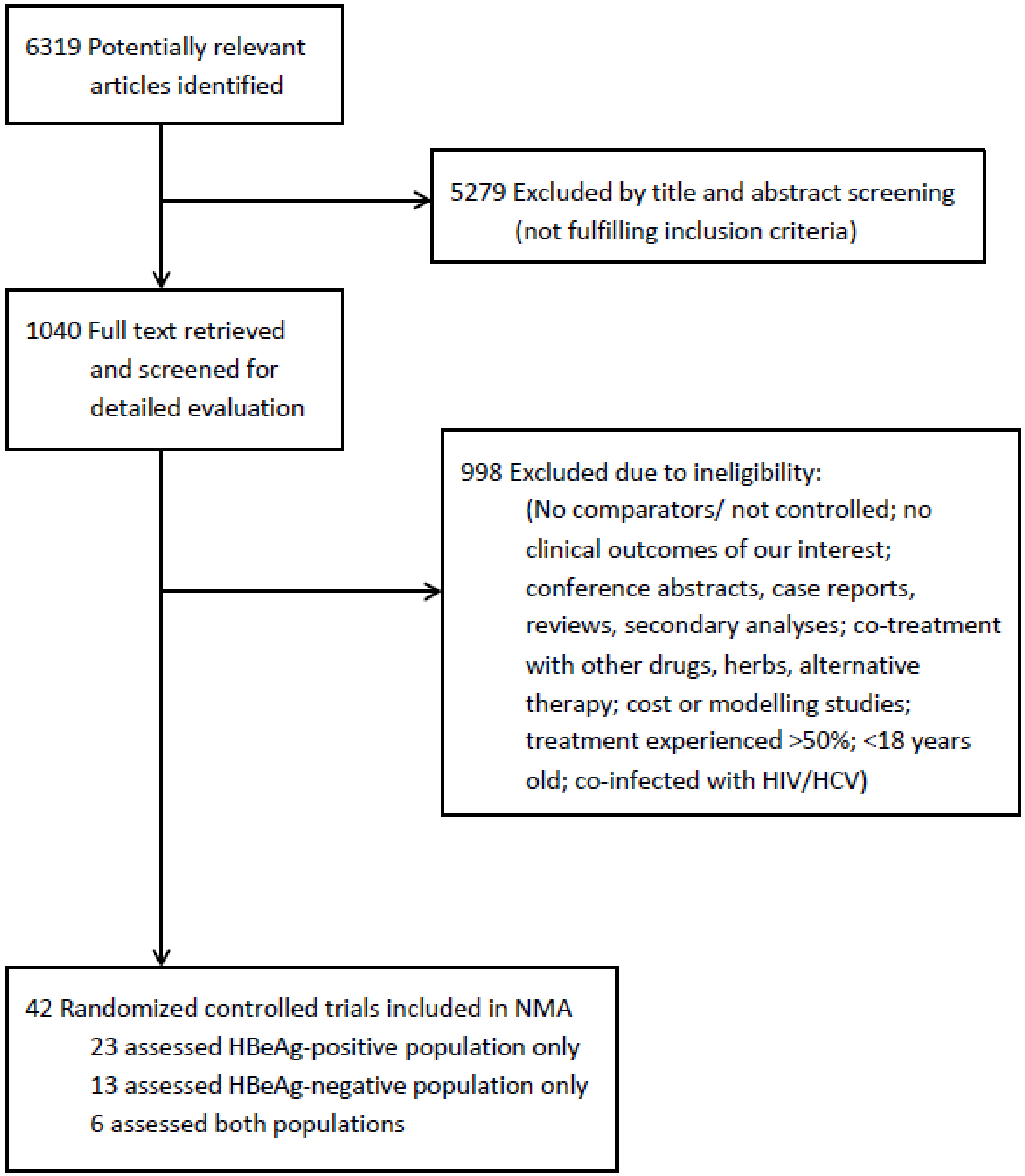

MEDLINE

Database(s): Database(s): Ovid MEDLINE: Epub Ahead of Print, In-Process & Other Non-Indexed Citations, Ovid MEDLINE® Daily and Ovid MEDLINE® 1946-Present

Filter: Cochrane Highly Sensitive Search Strategy for identifying randomized trials in MEDLINE: sensitivity- and precision-maximizing version (2008 revision); Ovid format from Higgins JPT, Green S (editors). Cochrane Handbook for Systematic Reviews of Interventions Version 5.1.0 [updated March 2011]. The Cochrane Collaboration, 2011. Available from www.cochrane-handbook.org.

Limits: Humans

Search Strategy:

#	Searches
1	interferon-alpha/ or ((interferon adj5 alpha) or (interferon adj5 alfa) or (peginterferon adj5 alpha) or (ifn adj5 alpha) or alpha-interferon or interferon-alpha or ifn-alpha or leif).mp. or (198153-51-4 or 215647-85-1).rn.
2	lamivudine/ or zidovudine/ or lamivudine.nm. or (epivir-hbv or "gr 103665" or gr103665 or "nsc 6207533" or "nsc6207533" or 3tc or bch189 or "bch 189" or gr109714x or "gr 109714x" or hepitec or heptovir or hepivir or heptodin or trizivir or zefix or zeffix or zidovudine or lamivudine or lamavudine or combivir or epzicom or epivir or inhavir or ladiwin or lamidac or lamivir or slamivudine).mp. or 134680-32-3.rn.
3	(adefovir or hepsera or preveon or pmea or adv or phoshonylmethoxyethyl: or "adefovir dipivoxil" or adefovirdipivoxl or "bis-pom pmea" or "bis(pom) pmea" or piv2pmea or "gs 0393" or gs0393 or gs840 or "gs 840" or gs0840 or "gs 0840").mp. or (106941-25-7 or 142340-99-6).rn.
4	(entecavir or baraclude or etv or "bms 200475" or bms200475 or "sq 34676" or sq34676 or telbivudin? or epavudine or "ldt 600" or ldt600 or ldt-600 or "nv 02b nv02b" or tyzeka or l-dt or sebivo or "tenofovir disoproxil fumarate" or tdf or pmpa or tenofovir$).mp. or (3424-98-4 or 147127-19-3 or 147127-20-6).rn. or (142217-69-4 or 209216-23-9).rn.
5	((Tenofovir adj2 (alafenamide or alafenamide-d5 or alagenamide)) or TAF or GS-7340 or "GS 7340" or GS7340 or 99YXE507IL or GS-7340-03 or GS734003 or GS-734003 or "GS 7340 03" or Vemlidy or Genvoya or Descovy or Odefsey or (NCT01780506 or NCT01797445 or NCT01818596 or NCT02121795 or NCT01815736)).mp. or ("379270 37 8" or "379270 38 9" or "731772 56 8" or "1392275 56 7" or "377091 31 1").rn.
6	1 or 2 or 3 or 4 or 5

7	hepatitis b/ or hepatitis b virus/ or hepatitis b, chronic/ or hepatitis b antibodies/ or hepatitis b antigens/ or hepatitis b core antigens/ or hepatitis b e antigens/ or hepatitis b surface antigens/ or ("hep b" or "hepatitis b" or "type b hepatitis" or "hbv" or ("chronic homologous" adj4 serum adj4 jaundice) or (chronic adj5 diffuse adj5 hepatocellular adj5 inflamm:) or (Anti adj HBAg) or HBAg or HBsAg or HBcAg).mp.
8	((randomized controlled trial or controlled clinical trial).pt. or (randomly or randomized or placebo?).ab. or clinical trials as topic.sh. or trial.ti.) not (exp animals/ not humans.sh.)
9	6 and 7 and 8
