# Supplementary material for: Antiviral treatment for treatment-naïve chronic hepatitis B: systematic review and network meta-analysis of randomized controlled trials
Source: Syst Rev. 2019 Aug 19;8:207. doi: 10.1186/s13643-019-1126-1 (PMC6699129; doi:10.1186/s13643-019-1126-1)
Supplement: Supplementary file 2 — Cochrane risk of bias assessment results. (PDF 375 kb) [file 13643_2019_1126_MOESM2_ESM.pdf]

Appendix B: Cochrane risk of bias assessment results

Author, Year	Random sequence generation	Allocation concealment	Blinding of participants and personnel	Blinding of outcome assessment	Complete-ness of outcome data	Free of selective reporting	Free of other bias
Bozkaya et al. 2005	?	?	?	?	+	+	?
Brouwer et al. 2015	+	+	-	+	+	+	+
Buti et al. 2016	+	+	+	?	+	+	+
Cao et al. 2013	?	?	?	+	+	+	+
Chan et al. 2007a	+	+	?	+	+	+	+
Chan et al. 2007b	+	+	+	+	+	+	+
Chan et al. 2016	+	+	+	+	+	+	+
Chang et al. 2006	?	+	+	+	+	+	+
Dienstag et al. 1999	+	?	+	?	+	+	+
Hadziyannis et al. 2003	+	+	+	+	+	+	+
He et al. 2012	+	?	?	+	+	+	+
Hou et al. 2015	+	+	+	+	+	+	+
Janssen et al. 2005	+	+	+	?	+	+	+
Jia et al. 2014	+	+	+	+	+	+	+
Kaymakoglu et al. 2007	+	?	?	+	+	+	?
Koike et al. 2017	?	+	+	+	+	+	+
Lai et al. 1998	+	+	+	?	+	+	+
Lai et al. 2005	?	+	+	+	+	+	+
Lai et al. 2006	?	+	+	?	+	+	+
Lampetico et al. 2013	+	+	?	?	+	+	+
Lau et al. 2005	+	+	+	+	+	+	+
Lee et al. 2017	+	+	+	?	+	+	+

Author, Year	Random sequence generation	Allocation concealment	Blinding of participants and personnel	Blinding of outcome assessment	Complete-ness of outcome data	Free of selective reporting	Free of other bias
Leung et al. 2009	?	?	?	?	+	+	+
Liang et al. 2015	?	?	–	?	+	+	+
Liaw et al. 2009	+	+	+	+	+	+	+
Liu et al. 2014	+	+	?	?	+	+	+
Lok et al. 2012	+	+	?	?	+	+	+
Marcellin et al. 2003	+	+	+	+	+	+	+
Marcellin et al. 2004	+	+	+	+	+	+	+
Marcellin et al. 2008	+	+	+	?	+	+	+
Marcellin et al. 2016	+	+	–	?	+	+	+
Papadopoulos et al. 2009	+	–	–	?	+	+	+
Piccolo et al. 2009	+	+	+	?	+	+	+
Ren et al. 2007	?	?	?	?	+	+	+
Sriprayoon et al. 2017	?	?	?	?	+	+	+
Sung et al. 2008	+	?	+	?	+	+	+
Tangkijvanich et al. 2016	+	?	–	?	+	+	+
Tassopoulos et al. 1999	+	?	+	?	+	+	+
Tseng et al. 2014	?	?	+	?	+	+	+
Xie et al. 2014	+	+	–	+	+	+	+
Yao et al. 2007	?	?	+	?	+	+	+
Zhang et al. 2016	+	?	–	?	+	+	+
