# Supplementary material for: Antiviral treatment for treatment-naïve chronic hepatitis B: systematic review and network meta-analysis of randomized controlled trials
Source: Syst Rev. 2019 Aug 19;8:207. doi: 10.1186/s13643-019-1126-1 (PMC6699129; doi:10.1186/s13643-019-1126-1)
Supplement: Supplementary file 3 — Characteristics of included randomized controlled trials. (PDF 358 kb) [file 13643_2019_1126_MOESM3_ESM.pdf]

Appendix C: Characteristics of Included RCTs

Author, Year, Design	Treatment/ follow-up Duration (weeks)	Sample size	Active Comparators	Treatment codes used in network meta-analysis	Outcomes reported
**HBeAg Positive**					
Brouwer et al. 2015<br>Open-label	96	175	1. ETV 0.5mg daily<br>2. ETV 0.5mg daily + Peg-IFN add-on (180 mg/week) from week 24 to 48	ETV<br>ETVPEG	HBV DNA, ALT norm, HBeAg loss, HBeAg sero, HBsAg loss
Cao et al. 2013	120	47	1. LAM 100mg daily + PEG IFNα-2a 135 mg weekly<br>2. ADV 10mg daily + PEG IFNα-2a 135 mg weekly	LAMPEG2<br>PEGADV1	HBV DNA, HBeAg sero, HBsAg loss
Chan et al. 2007a<br>Open-label	52	131	1. ADV 10mg daily<br>2. TBV 600mg daily<br>3. ADV 10mg daily for 24 weeks then switched to TBV 600mg daily	ADV<br>TBV<br>ADVTBV	HBV DNA, ALT norm, HBeAg loss, HBeAg sero, HBsAg loss
Chan et al. 2016<br>DB	48	873	1. TAF 25mg daily<br>2. TDF 300mg daily	TAF<br>TDF	HBV DNA, ALT norm, HBeAg loss, HBeAg sero, HBsAg loss
Chang et al. 2006<br>DB	48	709	1. LAM 100mg daily<br>2. ETV 0.5mg daily	LAM<br>ETV	HBV DNA, ALT norm, HBeAg loss, HBeAg sero, HBsAg loss
Dienstag et al. 1999<br>DB	68	137	1. PLA<br>2. LAM 100mg daily	PLA<br>LAM	HBeAg loss, HBeAg sero, HBsAg loss
He at al. 2012	96	137	1. LAM 100mg daily<br>2. ADV 10mg daily<br>3. LAM 100 mg + ADV 10 mg daily for 12-24wks followed by ADV alone	LAM<br>ADV<br>LAMADV	HBV DNA, ALT norm, HBeAg sero
Hou etal. 2015<br>DB	48	202	1. ADV 10mg daily<br>2. TDF 300mg daily	ADV<br>TDF	HBV DNA, ALT norm, HBeAg loss, HBeAg sero, HBsAg loss
Janssen et al. 2005<br>DB	78	266	1. PEGα-2b 100μg weekly (50μg weekly from wk 32)<br>2. LAM 100mg daily + PEGα-2b 100μg weekly (50μg weekly from wk 32)	PEG<br>LAMPEG	HBV DNA, ALT norm, HBeAg loss, HBeAg sero, HBsAg loss
Jia et al. 2014<br>DB	104	290	1. LAM 100mg daily<br>2. TBV 600mg daily	LAM<br>TBV	HBV DNA, ALT norm, HBeAg loss, HBeAg sero, HBsAg loss
Koike etal. 2017<br>DB	48	165	1. TDF 300mg daily<br>2. ETV 0.5mg daily	TDF<br>ETV	HBV DNA, ALT norm, HBeAg sero
Lai et al. 1998<br>DB	52	215	1. PLA<br>2. LAM 100mg daily	PLA<br>LAM	ALT norm, HBeAg sero, HBsAg loss

Author, Year, Design	Treatment/ follow-up Duration (weeks)	Sample size	Active Comparators	Treatment codes used in network meta-analysis	Outcomes reported
Lai et al. 2005<br>DB	52	104	1. LAM 100mg daily<br>2. TBV 400/600mg daily<br>3. LAM100mg + TBV 400/600mg daily	LAM<br>TBV<br>LAMTBV	HBV DNA, ALT norm, HBeAg loss, HBeAg sero, HBsAg loss
Lau et al. 2005<br>Partial DB	72	814	1. LAM 100mg daily<br>2. PEGα-2a 180µg weekly + PLA<br>3. LAM 100mg daily + PEGα-2a 180µg weekly	LAM<br>PEG<br>LAMPEG1	HBV DNA, ALT norm, HBeAg loss, HBeAg sero
Leung et al. 2009<br>Open-label	48	65	1. ADV 10mg daily<br>2. ETV 0.5mg daily	ADV<br>ETV	HBV DNA, ALT norm, HBeAg loss, HBeAg sero
Liang etal. 2015<br>Open-label	104	358	1. LAM 100 mg + ADV 10 mg daily<br>2. LAM 100 mg daily + add ADV 10 mg daily to suboptimal responders from wk 30<br>3. LAM 100 mg	LAMADV1<br>LAMADV2<br>LAM	HBV DNA, ALT norm, HBeAg loss, HBeAg sero, HBsAg loss
Liaw et al. 2009<br>DB	104	921	1. LAM 100mg daily<br>2. TBV 600mg daily	LAM<br>TBV	HBV DNA, ALT norm, HBeAg loss, HBeAg sero, HBsAg loss
Liu et al. 2014<br>Open-label	52	61	1. PEGα-2b 1.5µg/kg weekly<br>2. PEGα-2b 1.5µg/kg weekly + ADV 10mg daily	PEG<br>PEGADV	HBV DNA, ALT norm, HBsAg loss
Lok et al. 2012<br>Open-label	96	264	1. ETV 0.5mg daily<br>2. ETV 0.5mg daily + TDF 300mg daily	ETV<br>ETVTDF	HBV DNA, ALT norm, HBeAg loss, HBeAg sero, HBsAg loss
Marcellin et al. 2003<br>DB	48	511	1. PLA<br>2. ADV 10mg daily<br>3. ADV 30mg daily	PLA<br>ADV<br>ADV2	HBV DNA, ALT norm, HBeAg loss, HBeAg sero
Marcellin et al. 2008<br>DB	48	266	1. ADV 10mg daily<br>2. TDF 300mg daily	ADV<br>TDF	HBV DNA, ALT norm, HBeAg sero, HBsAg loss
Marcellin etal. 2016<br>Open-label	72	740	1. TDF 300mg daily + PEG α-2a 180µg weekly for 48 wks<br>2. TDF 300mg daily for 48 wks + PEG α-2a 180µg weekly for 1st 16 wks<br>3. TDF 300mg daily for 72 wks<br>4. PEG α-2a 180µg weekly for 48 wks	TDFPEG1<br>TDFPEG2<br>TDF<br>PEG	HBV DNA, ALT norm, HBeAg loss, HBeAg sero, HBsAg loss
Ren et al. 2007	48	42	1. LAM 100mg daily<br>2. ETV 0.5mg daily	LAM<br>ETV	HBV DNA, ALT norm, HBeAg sero
Sriprayoon etal. 2017	144	400	1. ETV 0.5mg daily<br>2. TDF 300mg daily	ETV<br>TDF	HBV DNA, ALT norm, HBeAg loss, HBeAg sero, HBsAg loss
Sung et al. 2008<br>DB	104	111	1. LAM 100mg daily<br>2. LAM 100 mg + ADV 10 mg daily	LAM<br>LAMADV1	HBV DNA, ALT norm, HBeAg loss, HBeAg sero
Tseng etal. 2014	52	42	1. ETV: 0.5mg daily	ETV	HBeAg loss, HBeAg sero

Author, Year, Design	Treatment/ follow-up Duration (weeks)	Sample size	Active Comparators	Treatment codes used in network meta-analysis	Outcomes reported
			2. PLA	PLA	
Xie etal. 2014<br>Open-label	72	218	1. PEG α-2a 180μg weekly for 48 wks<br>2. PEG α-2a 180μg weekly for 48 wks + ETV add-on at week 13 for 24 weeks<br>3. ETV 0.5mg daily for 24 wks + PEG α-2a from week 21 for 48 wks	PEG<br>PEGETV<br>ETVPEG1	HBV DNA, ALT norm, HBeAg loss, HBeAg sero, HBsAg loss
Yao et al. 2007<br>DB	48	446	1. LAM 100mg daily<br>2. ETV 0.5mg daily	LAM<br>ETV	HBV DNA, ALT norm, HBeAg loss, HBeAg sero
Zhang etal. 2016<br>Open-label	96	129	1. PEGα-2a 135 μg weekly for 48 wks<br>2. PEGα-2a 135 μg weekly + ADV 10mg daily add-on at wk 0<br>3. PEGα-2a 135 μg weekly + ADV 10mg daily add-on at wk 12<br>4. PEGα-2a 135 μg weekly + ADV 10mg daily add-on at wk 24	PEG<br>PEGADV1<br>PEGADV2<br>PEGADV3	HBV DNA, ALT norm, HBeAg sero, HBsAg loss
**HBeAg Negative**					
Bozkaya et al. 2005	52	55	1. No treatment<br>2. LAM 100mg daily	PLA<br>LAM	ALT norm
Buti etal. 2016<br>DB	48	425	1. TAF 25mg daily<br>2. TDF 300mg daily	TAF<br>TDF	HBV DNA, ALT norm
Chan et al. 2007b<br>DB	120	136	1. PLA<br>2. LAM 100mg daily	PLA<br>LAM	HBV DNA, ALT norm
Hadziyannis et al. 2003<br>DB	48	184	1. PLA<br>2. ADV 10mg daily	PLA<br>ADV	HBV DNA, ALT norm
Hou etal. 2015<br>DB	48	307	1. ADV 10mg daily<br>2. TDF 300mg daily	ADV<br>TDF	HBV DNA, ALT norm
Jia et al. 2014<br>DB	104	42	1. LAM 100mg daily<br>2. TBV 600mg daily	LAM<br>TBV	HBV DNA, ALT norm
Kaymakoglu et al. 2007<br>Open-label	72	48	1. PEGα-2b 1.5μg/kg weekly<br>2. LAM 100mg daily + PEGα-2b 1.5μg/kg weekly	PEG<br>LAMPEG1	HBV DNA, ALT norm
Lai et al. 2006<br>DB	48	638	1. LAM 100mg daily<br>2. ETV 0.5mg daily	LAM<br>ETV	HBV DNA, ALT norm

Author, Year, Design	Treatment/ follow-up Duration (weeks)	Sample size	Active Comparators	Treatment codes used in network meta-analysis	Outcomes reported
Lampetico et al. 2013<br>Open-label	96	128	1. PEGα-2a 180μg weekly for 48 wks<br>2. PEGα-2a 180μg weekly for 48 wks then 135μg weekly for 48 wks<br>3. (LAM 100mg + PEGα-2a 180μg weekly) for 48 wks then PEG 135μg weekly for 48 wks	PEG<br>PEGPEG<br>LAMPEG	HBV DNA, ALT norm
Lee etal. 2017<br>Partial DB	240	120	1. ETV 0.5mg daily<br>2. LAM 100mg daily	ETV<br>LAM	HBV DNA
Liaw et al. 2009<br>DB	104	446	1. LAM 100mg daily<br>2. TBV 600mg daily	LAM<br>TBV	HBV DNA, ALT norm
Lok et al. 2012<br>Open-label	96	115	1. ETV: 0.5mg daily<br>2. ETV 0.5mg daily + TDF 300mg daily	ETV<br>ETVTDF	HBV DNA, ALT norm
Marcellin et al. 2004<br>Partial DB	72	537	1. LAM 100mg daily<br>2. PEGα-2a 180μg weekly<br>3. LAM 100mg daily + PEGα-2a 180μg weekly	LAM<br>PEG<br>LAMPEG1	HBV DNA, ALT norm
Marcellin et al. 2008<br>DB	48	375	1. ADV 10mg daily<br>2. TDF 300mg daily	ADV<br>TDF	HBV DNA, ALT norm
Papadopoulos et al. 2009	72	123	1. PEGα-2b 1.5μg/kg weekly<br>2. LAM 100mg daily + PEGα-2b 1.5μg/kg weekly	PEG<br>LAMPEG1	HBV DNA, ALT norm
Piccolo et al. 2009<br>DB	72	60	1. PEGα-2a 1.5μg/kg weekly<br>2. PEGα-2a 1.5μg/kg weekly + ADV 10mg/day	PEG<br>PEGADV	HBV DNA, ALT norm
Tangkijvanich etal. 2016<br>Open-label	96	126	1. PEGα-2b 1.5μg/kg weekly<br>2. ETV 0.5mg daily + PEGα-2b 1.5μg/kg weekly	PEG<br>PEGETV	HBV DNA, ALT norm
Tassopoulos et al. 1999<br>DB	52	108	1. PLA<br>2. LAM 100mg daily	PLA<br>LAM	HBV DNA, ALT norm
Yao et al. 2007<br>DB	48	73	1. LAM 100mg daily<br>2. ETV 0.5mg daily	LAM<br>ETV	HBV DNA, ALT norm

Abbreviations: DB, double-blind; ADV, adefovir; ETV, entecavir; LAM, lamivudine; PEG, pegylated interferon; PLA, placebo; TAF, tenofovir alafenamide; TBV, telbivudine; TDF, tenofovir disoproxil fumarate; HBV DNA, undetectable HBV DNA levels; ALT norm, normalization of serum alanine aminotransferase levels; HBeAg sero, hepatitis B e antigen seroconversion; HBeAg loss, hepatitis B e antigen loss; HBsAg loss, hepatitis B surface antigen loss
