# Supplementary material for: Antiviral treatment for treatment-naïve chronic hepatitis B: systematic review and network meta-analysis of randomized controlled trials
Source: Syst Rev. 2019 Aug 19;8:207. doi: 10.1186/s13643-019-1126-1 (PMC6699129; doi:10.1186/s13643-019-1126-1)
Supplement: Supplementary file 4 — Participant baseline characteristics of included randomized controlled trials. (PDF 398 kb) [file 13643_2019_1126_MOESM4_ESM.pdf]

Appendix D: Participant Baseline Characteristics of included RCTs

Author, Year, Design	Mean Age ±SD/ Median Age (Range)	Male (%)	Active Comparator	Baseline HBV DNA	Baseline ALT
**HBeAg Positive**					
Brouwer et al. 2015<br>Open-label	31±9<br>32±10	69<br>74	• ETV 0.5mg daily<br>• ETV 0.5mg daily + Peg-IFN add-on (180 mg/week) from week 24 to 48	7.8±1.1<br>7.8±1.3<br>(logIU/ml, mean±SD)	2.7±2.1<br>3.1±3.3<br>(mean±SD)
Cao et al. 2013	32.3±12<br>35.7±9.4	71<br>74	• LAM 100mg daily + PEG IFNα-2a 135 mg weekly<br>• ADV 10mg daily + PEG IFNα-2a 135 mg weekly	7.3 ±1.1<br>7.1 ±1.1<br>(log copies/mL, mean± SD)	144.2 ±64.5<br>149.8 ±62.7<br>(U/L, mean ±SD)
Chan et al. 2007a<br>Open-label	30 (19-47)<br>34 (18-60)<br>33 (18-53)	91<br>78<br>59	• ADV 10mg daily<br>• TBV 600mg daily<br>• ADV 10mg daily for 24 weeks then switched to TBV 600mg daily	9.98 ±0.23<br>9.57 ±0.26<br>9.47 ±0.29<br>(log_{10} copies/mL , mean ± SE)	199±25.7<br>183±23.6<br>138±12.8<br>(U/L, mean±SE)
Chan et al. 2016<br>DB	38±11<br>38±12	64<br>65	• TAF 25mg daily<br>• TDF 300mg daily	7.6±1.34<br>7.6±1.41<br>(log_{10} IU/ml, mean±SD)	117±105.1<br>125±128.2<br>(U/L, mean±SD)
Chang et al. 2006<br>DB	35 ±13<br>35 ±13	74<br>77	• LAM 100mg daily<br>• ETV 0.5mg daily	9.69 ±1.99<br>9.62 ±2.01<br>(log copies/ml, mean ±SD)	146.3 ±123.3<br>140.5 ±114.3<br>(IU/L, mean ±SD)
Dienstag et al. 1999<br>DB	38(20-67)<br>40(18-73)	80<br>86	• PLA<br>• LAM 100mg daily	56.5 (0.8-653)<br>102.2 (0.8-1753)<br>pg/ml, median (range)	135 (33-592)<br>125 (46-401)<br>U/L, median (range)
He at al. 2012	38.9 ± 6.7<br>38.6 ± 6.6<br>39.6 ± 7.2	72<br>70<br>76	• LAM 100mg daily<br>• ADV 10mg daily<br>• LAM 100 mg + ADV 10 mg daily for 12-24wks followed by ADV alone	8.2 ± 0.9<br>8.1 ± 0.7<br>8.3 ± 0.8<br>(log_{10} copies/ml, mean±SD)	4.5 ± 2.1<br>5.3 ± 2.2<br>5.2 ± 2.2<br>(xULN, mean±SD)
Hou etal. 2015<br>DB	36.4(18-66)<br>36.1(18-66)	83.3<br>83.3	• ADV 10mg daily<br>• TDF 300mg daily	8.7 ± 0.79<br>8.7 ± 0.87<br>(log_{10} copies/ml, mean±SD)	189 ± 121.5<br>199.1 ± 132.8<br>(U/L, mean±SD)
Janssen et al. 2005<br>DB	36 ± 14<br>34 ± 12	79<br>75	• PEGα-2b 100μg weekly (reduced to 50μg from wk 32)<br>• LAM 100mg daily + PEGα-2b 100μg weekly (reduced to 50μg from wk 32)	9.1 ±0.8<br>9.1 ±1<br>(log copies/ml, mean ±SD)	4.3±3.1<br>4.4±3.9<br>(xULN, mean±SD)
Jia et al. 2014<br>DB	29.9 (15-63)<br>29.3 (16-64)	76.4<br>80.8	• LAM 100mg daily<br>• TBV 600mg daily	9.4 ± 0.14<br>9.2 ± 0.12<br>(log_{10} copies/ml, mean±SE)	159.3 ± 14.72<br>157 ± 8.92<br>(IU/L, mean ±SE)

Author, Year, Design	Mean Age ±SD/ Median Age (Range)	Male (%)	Active Comparator	Baseline HBV DNA	Baseline ALT
Koike etal. 2017<br>DB	45 (22-69)<br>46.5 (30-67)	62<br>71	• TDF 300mg daily<br>• ETV 0.5mg daily	7±1.5<br>7.19±1.3<br>(log_{10} copies/ml, mean±SD)	90.4±99.1<br>76.7±80.7<br>(IU/L, mean ±SD)
Lai et al. 1998<br>DB	29 (15-67)<br>31 (16-55)	72<br>74	• PLA<br>• LAM 100mg daily	99.4 (1-990)<br>74.2 (1-516)<br>pg/ml, median (range)	1.5 (0-10)<br>1.5 (0-15)<br>xULN, meaian (range)
Lai et al. 2005<br>DB	34 (18-61)<br>41 (19-68)<br>32 (19-53)	74<br>79.5<br>85	• LAM 100mg daily<br>• TBV 400/600mg daily<br>• LAM100mg + TBV 400/600mg daily	9.3 (6.6-12.9)<br>9.0 (6.3-13.3)<br>9.5 (5.9-13.2)<br>log_{10} copies/ml, median (range)	122 (62-309)<br>130 (35-400)<br>142 (32-1657)<br>U/L, median (range)
Lau et al. 2005<br>Partial DB	30 (17-65)<br>31 (18-77)<br>29 (18-66)	79<br>79<br>77	• LAM 100mg daily<br>• PEGα-2a 180µg weekly + PLA<br>• LAM 100mg daily + PEGα-2a 180µg weekly	10.1±2.0<br>9.9±2.1<br>10.1±1.9<br>(log copies/ml, mean±SD)	102.3±78.4<br>114.6±114.3<br>114.9±94.1<br>(IU/L, mean ±SD)
Leung et al. 2009<br>Open-label	32 ± 2<br>37 ± 2.4	66<br>61	• ADV 10mg daily<br>• ETV 0.5mg daily	9.88 ± 0.22<br>10.26 ± 0.35<br>(log_{10} copies/ml, mean±SE)	172.3 ± 37<br>110.6 ± 14.6<br>(U/L, mean ±SE)
Liang etal. 2015<br>Open-label	30(18-58)<br>28(18-59)<br>31(18-63)	78.3<br>77.5<br>75.4	• LAM 100 mg + ADV 10 mg daily<br>• LAM 100 mg daily + ADV 10 mg daily to suboptimal responders from wk 30<br>• LAM 100 mg	8.6±0.9<br>8.6±0.9<br>8.6±0.9<br>(log_{10} copies/ml, mean±SD)	3.3±2.7<br>3.4±2.1<br>3.6±2.8<br>(xULN, mean ±SD)
Liaw et al. 2009<br>DB	33 (16-67)<br>32 (16-63)	76<br>73	• LAM 100mg daily<br>• TBV 600mg daily	9.5 ± 0.1<br>9.5 ± 0.1<br>(log_{10} copies/ml, mean±SE)	158.9 ± 6.3<br>146.4 ± 5.37<br>(IU/L, mean ±SE)
Liu et al. 2014<br>Open-label	24 (16-39)<br>27 (21-44)	68<br>63	• PEGα-2b 1.5µg/kg weekly<br>• ADV 10mg daily + PEGα-2b 1.5µg/kg weekly	7.51 (6.13-8.88)<br>7.37 (5.61-8.01)<br>log U/ml, median (range)	4.1 (2.33-9.95)<br>3.83 (1.78-9.25)<br>xULN, median (range)
Lok et al. 2012<br>Open-label	40 ± 1.1<br>39 ± 1	63.7<br>74.1	• ETV 0.5mg daily<br>• ETV 0.5mg daily + TDF 300mg daily	8.1 ± 0.09<br>8.15 ± 0.08<br>(log_{10} IU/ml, mean±SE)	127 ± 7.3<br>158 ± 13.1<br>(U/L, mean ±SE)
Marcellin et al. 2003<br>DB	35 (16-66)<br>32 (16-65)<br>32 (17-68)	71<br>76<br>75	• PLA<br>• ADV 10mg daily<br>• ADV 30mg daily	8.12±0.89<br>8.25±0.90<br>8.22±0.84<br>(log copies/ml, mean±SD)	139±131<br>139±154<br>124±96<br>(U/L, mean ±SD)
Marcellin et al. 2008<br>DB	34 ± 12<br>34 ± 11	71<br>68	• ADV 10mg daily<br>• TDF 300mg daily	8.88 ±0.93<br>8.64 ±1.076<br>(log_{10} copies/ml, mean±SD)	155±121.49<br>142±102.81<br>(IU/L, mean ±SD)

Author, Year, Design	Mean Age ±SD/ Median Age (Range)	Male (%)	Active Comparator	Baseline HBV DNA	Baseline ALT
Marcellin etal. 2016<br>Open-label	38±16.7<br>37±9<br>36±10.9<br>38±10.5	68<br>65<br>65<br>64	• TDF 300mg daily + PEG α-2a 180µg weekly for 48 wks<br>• TDF 300mg daily + PEG α-2a 180µg weekly for 16 wks followed by TDF only for 32 wks<br>• TDF 300mg daily<br>• PEG α-2a 180µg weekly for 48 wks	7.1±1.5<br>7.1±1.5<br>7.0±1.5<br>6.9±1.6<br>(log_{10} IU/ml, mean±SD)	121±181<br>112±94<br>101±68<br>107±92<br>(U/L, mean ±SD)
Ren et al. 2007	31±12<br>33±10	52<br>57	• LAM 100mg daily<br>• ETV 0.5mg daily	8.49 ± 1.10<br>8.52 ± 1.02<br>(log copies/ml, mean±SD)	201.6 ± 178.2<br>211.2 ± 144.7<br>(IU/L, mean ±SD)
Sriprayoon etal. 2017	41.6±11.5<br>41.2±11.6	60.5<br>56.5	• ETV 0.5mg daily<br>• TDF 300mg daily	7.1±1.5<br>7.0±1.3<br>(log_{10} IU/ml, mean±SD)	68.1±64.1<br>76.8±79.8<br>(U/L, mean ±SD)
Sung et al. 2008<br>DB	36 (18-79)<br>33 (17-63)	74<br>83	• LAM 100mg daily<br>• LAM 100 mg + ADV 10 mg daily	9.17 (4.4-11.1)<br>8.87 (6.5-11.0)<br>log_{10} copies/ml, median (range)	-
Tseng etal. 2014	45±10<br>42±12	59<br>55	• ETV: 0.5mg daily<br>• PLA	5.95±1.3<br>6.31±1.42<br>(log copies/ml, mean±SD)	0.6±0.2<br>0.6±0.2<br>(xULN, mean ±SD)
Xie etal. 2014<br>Open-label	29.5±8.1<br>29.2±6.9<br>30±8.4	77.8<br>78.1<br>82.2	• PEG α-2a 180µg weekly for 48 wks<br>• PEG α-2a 180µg weekly for 48 wks + 24 weeks of ETV add-on at week 13<br>• ETV 0.5mg daily for 24 wks + PEG α-2a at week 21 for 48 wks	7.1±0.8<br>7.1±1.2<br>7.1±1.1<br>(log copies/ml, mean±SD)	4.1±2.5<br>3.8±2.1<br>4.3±3.4<br>(xULN, mean ±SD)
Yao et al. 2007<br>DB	30±9<br>30±9	83<br>82	• LAM 100mg daily<br>• ETV 0.5mg daily	8.65 ± 1<br>8.77 ± 0.86<br>(log copies/ml, mean±SD)	204 ± 192<br>191 ± 135<br>(U/L, mean ±SD)
Zhang etal. 2016<br>Open-label	28.56±7.95<br>30.15±7.19<br>29.5±8.08<br>29.28±6.94	78.1<br>78.8<br>75<br>81.3	• PEGα-2a 135 µg weekly for 48 wks<br>• PEGα-2a 135 µg weekly + ADV 10mg daily add-on at wk 0<br>• PEGα-2a 135 µg weekly + ADV 10mg daily add-on at wk 12<br>• PEGα-2a 135 µg weekly + ADV 10mg daily add-on at wk 24	7.15±1.14<br>6.78±1.35<br>7.03±1.21<br>7.08±1.04<br>(log copies/ml, mean±SD)	4.3±2.6<br>3.9±2.4<br>4.1±2.7<br>3.7±2.4<br>(xULN, mean ±SD)
**HBeAg Negative**					

Author, Year, Design	Mean Age ±SD/ Median Age (Range)	Male (%)	Active Comparator	Baseline HBV DNA	Baseline ALT
Bozakaya et al. 2005	39.0 ±7.2 31.9 ±11.1	68<br>94	• PLA (No treatment)<br>• LAM 100mg daily	4.2×10^3 (1×10^2–3.6×10^5)<br>1.2 x10^3<br>copies/ml, median (range)	48 (35–168)<br>63.5 (38–186)<br>IU/L, median (range)
Buti etal. 2016<br>DB	45±12<br>48±10	61<br>61	• TAF 25mg daily<br>• TDF 300mg daily	5.7±1.3<br>5.8±1.3<br>(log_{10} IU/ml, mean±SD)	67(44-102)<br>67(47-102)<br>U/L, median (IQR)
Chan et al. 2007b<br>DB	39 ± 11<br>39 ±10	83<br>84	• PLA<br>• LAM 100mg daily	5.6 ± 1.5<br>5.7 ± 1.6<br>(log copies/ml, mean±SD)	2.6±2.3<br>2.1±1.7<br>(xULN, mean ±SD)
Hadziyannis et al. 2003<br>DB	45±10.4<br>46±9.8	82<br>83	• PLA<br>• ADV 10mg daily	6.9 ±1.0<br>6.9 ±0.9<br>(log copies/ml, mean±SD)	149.9±195.2<br>143.5±125.3<br>(U/L, mean ±SD)
Hou etal. 2015<br>DB	36.4(18-66)<br>36.1(18-66)	83.3<br>83.3	• ADV 10mg daily<br>• TDF 300mg daily	7.0 ± 1.13<br>6.9 ± 1.18<br>(log_{10} copies/ml, mean±SD)	112.6±80.3<br>133.4±120.9<br>(U/L, mean ±SD)
Jia et al. 2014<br>DB	36(19-58)<br>38(20-56)	86.4<br>85	• LAM 100mg daily<br>• TBV 600mg daily	7.5 ± 0.3<br>7.8 ± 0.4<br>(log_{10} copies/ml, mean±SE)	177±75.2<br>162±23.9<br>(IU/L, mean ±SE)
Kaymakoglu et al. 2007<br>Open-label	42.6±10.9<br>43± 7.8	68.4<br>69	• PEGα-2b 1.5μg/kg weekly<br>• LAM 100mg daily + PEGα-2b 1.5μg/kg weekly	182.3 ±175.4<br>209.6 ± 207.8<br>(pg/ml, mean±SD)	130.4±45<br>161.5±127.4<br>(IU/L, mean ±SD)
Lai et al. 2006<br>DB	44±11<br>44±11	75<br>76	• LAM 100mg daily<br>• ETV 0.5mg daily	7.6 ±1.7<br>7.6 ±1.8<br>(log copies/ml, mean±SD)	143±119.4<br>141±114.7<br>(IU/L, mean ±SD)
Lampetico et al. 2013<br>Open-label	45±10.2<br>44±10.4<br>46±8.6	65<br>87<br>72	• PEGα-2a 180μg weekly for 48 wks<br>• PEGα-2a 180μg weekly for 48 wks then 135μg weekly for 48 wks<br>• (LAM 100mg + PEGα-2a 180μg weekly) for 48 wks then PEG 135μg weekly for 48 wks	6.1 (2.1->8.0)<br>6.0 (2.6->8.0)<br>6.2 (3.5->8.0)<br>log_{10} IU/ml, median (range)	119 (34-897)<br>146 (51-897)<br>161 (34-897)<br>IU/L, median (range)
Lee etal. 2017<br>DB for 96 wks then open-label	45.7±11.9<br>49.0±7.8	84<br>75	• ETV 0.5mg daily<br>• LAM 100mg daily	6.1±0.8<br>5.8±0.9<br>(log_{10} copies/ml, mean±SD)	110.5±82.0<br>93.9±58.5<br>(IU/L, mean ±SD)
Liaw et al. 2009<br>DB	43 (18-68)<br>43 (17-68)	79<br>78	• LAM 100mg daily<br>• TBV 600mg daily	7.4±0.1<br>7.7±0.1<br>(log_{10} copies/ml, mean±SE)	143.7 ± 8.7<br>137 ± 6.94<br>(IU/L, mean ±SE)

Author, Year, Design	Mean Age ±SD/ Median Age (Range)	Male (%)	Active Comparator	Baseline HBV DNA	Baseline ALT
Lok et al. 2012<br>Open-label	40 ± 1.1<br>39 ± 1	63.7<br>74.1	• ETV: 0.5mg daily<br>• ETV 0.5mg daily + TDF 300mg daily	6.09 ± 0.18<br>6.09 ± 0.15<br>(log_{10} IU/ml, mean±SE)	127 ± 7.3<br>158 ± 13.1<br>(U/L, mean ±SE)
Marcellin et al. 2004<br>Partial DB	40±11.1<br>40±11.7<br>41±10.8	86<br>85<br>82	• LAM 100mg daily<br>• PEGα-2a 180μg weekly<br>• LAM 100mg daily + PEGα-2a 180μg weekly	7.24±1.78<br>7.14±1.84<br>7.35±2.00<br>(log copies/ml, mean±SD)	105.7±128.2<br>84.4±85.9<br>90.8±76.2<br>(IU/L, mean ±SD)
Marcellin et al. 2008<br>DB	43±10.0<br>44±10.6	78<br>77	• ADV 10mg daily<br>• TDF 300mg daily	6.98 ±1.27<br>6.86 ±1.31<br>(log_{10} copies/ml, mean±SD)	163.6±146.02<br>127.5±101.21<br>(IU/ml, mean ±SD)
Papadopoulos et al. 2009	46.3<br>46.7	86<br>74	• PEGα-2b 1.5μg/kg weekly<br>• LAM 100mg daily + PEGα-2b 1.5μg/kg weekly	6.16<br>5.78<br>(mean log_{10} copies/ml)	96.5<br>135.7<br>(mean IU/ml)
Piccolo et al. 2009<br>DB	45.9 ±10.2<br>48.3 ±10.7	60<br>73	• PEGα-2a 1.5μg/kg weekly<br>• PEGα-2a 1.5μg/kg weekly + ADV 10mg/day	5.7 ±0.9<br>5.9 ±1.0<br>(log_{10} IU/ml, mean±SD)	3.18±3.3<br>3.43±2.7<br>(xULN, mean±SD)
Tangkijvanich etal. 2016<br>Open-label	40.0±9.3<br>40.3±9.8	68.3<br>73	• PEGα-2b 1.5μg/kg weekly<br>• ETV 0.5mg daily + PEGα-2b 1.5μg/kg weekly	5.5±0.8<br>5.4±0.8<br>(log_{10} IU/ml, mean±SD)	75.5±32.2<br>72.5±40.0<br>(U/L, mean ±SD)
Tassopoulos et al. 1999<br>DB	44 (17-63)<br>42 (24-65)	77<br>83	• PLA<br>• LAM 100mg daily	95.5 (1.3-3,900)<br>255 (1.3-18,000)<br>pg/ml, median (range)	3.3 (0.7-12.5)<br>3.2 (0.6-16.4)<br>xULN, median (range)
Yao et al. 2007<br>DB	30±9<br>30±9	83<br>82	• LAM 100mg daily<br>• ETV 0.5mg daily	7.59 ±1.33<br>7.7 ±1.28<br>(log copies/ml, mean±SD)	164 ± 83<br>225 ± 169<br>(U/L, mean ±SD)

Abbreviations: DB, double-blind; ADV, adefovir; ETV, entecavir; LAM, lamivudine; PEG, pegylated interferon; PLA, placebo; TAF, tenofovir alafenamide; TBV, telbivudine; TDF, tenofovir disoproxil fumarate; HBV DNA, undetectable HBV DNA levels; ALT norm, normalization of serum alanine aminotransferase levels; HBeAg sero, hepatitis B e antigen seroconversion; HBeAg loss, hepatitis B e antigen loss; HBsAg loss, hepatitis B surface antigen loss
