# Supplementary material for: Antiviral treatment for treatment-naïve chronic hepatitis B: systematic review and network meta-analysis of randomized controlled trials
Source: Syst Rev. 2019 Aug 19;8:207. doi: 10.1186/s13643-019-1126-1 (PMC6699129; doi:10.1186/s13643-019-1126-1)
Supplement: Supplementary file 5 — Network inconsistency evaluation. (PDF 59 kb) [file 13643_2019_1126_MOESM5_ESM.pdf]

Appendix E: Network inconsistency evaluation

Inconsistency tests evaluate the validity of the consistency assumption in a network meta-analysis by assessing the compatibility of direct and indirect evidence. If the results from direct evidence conflict against the results from indirect evidence, then this highlights a problem of inconsistency in the network. In this case, one could argue that the direct and indirect evidence may not be compatible.

To evaluate inconsistency in the networks, we used a node-splitting method that excludes evidence from multi-arm studies. This method tests for the presence of sufficient evidence in the network to indicate the presence of inconsistency. Therefore, in the tables below any p-values below 0.05 would indicate that the difference in effect size between direct and indirect evidence is statistically significant from zero, and therefore inconsistency exists in the model. Absence of inconsistency tests for some of the outcomes can be attributed to the presence of a large number of multi-arm studies for that outcome. The results below show indicate that inconsistency was detected in some of the comparators that were evaluated. Care should be taken in the interpretation of the findings, as inconsistency tests are usually underpowered, due to small number of studies or events. Therefore, the absence of statistical significance does not necessarily suggest consistency but rather absence of sufficient evidence to indicate inconsistency.

**HBeAg positive population**								
		Direct Estimate		Indirect Estimate		NMA estimate		Inconsistency
Virological Response								
**T1**	**T2**	**OR**	**95% CI**	**OR**	**95% CI**	**OR**	**95% CI**	**p-value**
ADV	ETV	1.85	0.01 - 3.77	1.65	0.38 - 2.97	1.7	0.7 - 2.71	0.851
ADV	LDT	0.82	-0.88 - 2.54	1.4	-0.25 - 3.06	1.12	-0.03 - 2.26	0.607
ADV	TDF	2.9	1.77 - 4.06	2.63	0.9 - 4.34	2.81	1.92 - 3.73	0.773
**ETV**	**LAM**	**-1**	**-1.56 - -0.46**	**-3.05**	**-4.19 - -1.84**	**-1.42**	**-2.24 - -0.67**	**0.01**
**ETV**	**TDF**	**0.51**	**-0.34 - 1.35**	**2.25**	**1.09 - 3.25**	**1.12**	**0.22 - 1.99**	**0.02**
LAM	LDT	0.92	0.02 - 1.86	0.36	-1.87 - 2.57	0.84	0.04 - 1.67	0.606
**LAM**	**PEG**	**1.15**	**0.19 - 2.14**	**-1.38**	**-2.59 - -0.12**	**0.19**	**-0.98 - 1.39**	**0.006**
**PEG**	**TDF**	**3.26**	**2.34 - 4.23**	**0.73**	**-0.48 - 1.99**	**2.35**	**1.18 - 3.5**	**0.006**
ALT norm								

ADV	ETV	0.65	-0.93 - 2.26	0.44	-0.57 - 1.46	0.5	-0.31 - 1.33	0.821
ADV	LDT	-0.48	-2.13 - 1.12	0.49	-0.71 - 1.78	0.15	-0.8 - 1.09	0.315
ADV	PLA	-1.61	-2.81 - -0.41	-2.7	-4.37 - -1.07	-1.98	-2.97 - -1.05	0.257
ADV	TDF	0.42	-0.55 - 1.37	0.64	-0.8 - 1.94	0.49	-0.28 - 1.2	0.78
ETV	LAM	-0.57	-1.25 - 0.07	-1.7	-2.83 - -0.52	-0.85	-1.53 - -0.22	0.099
ETV	TDF	-0.38	-1.3 - 0.47	0.61	-0.56 - 1.59	-0.01	-0.78 - 0.69	0.156
LAM	LDT	0.62	-0.09 - 1.42	-0.34	-2.27 - 1.53	0.5	-0.17 - 1.19	0.322
**LAM**	**PEG**	**0.58**	**-0.09 - 1.25**	**-1.2**	**-2.13 - -0.19**	**-0.02**	**-0.97 - 0.88**	**0.012**
LAM	PLA	-2.11	-3.48 - -0.77	-1.02	-2.54 - 0.55	-1.63	-2.65 - -0.64	0.262
**PEG**	**TDF**	**1.52**	**0.8 - 2.25**	**-0.26**	**-1.13 - 0.73**	**0.87**	**-0.07 - 1.79**	**0.012**
HBeAg loss								
ADV	ETV	-0.23	-1.57 - 1.03	0.16	-0.53 - 0.86	0.07	-0.55 - 0.68	0.602
ADV	LDT	0.48	-0.6 - 1.57	0.39	-0.39 - 1.18	0.4	-0.21 - 1.04	0.89
ADV	PLA	-1	-1.75 - -0.28	-0.78	-1.91 - 0.32	-0.94	-1.56 - -0.33	0.737
ADV	TDF	0.65	-0.26 - 1.62	0.24	-0.64 - 1.12	0.44	-0.21 - 1.08	0.506
ETV	LAM	-0.04	-0.44 - 0.37	0.21	-0.69 - 1.08	0	-0.36 - 0.36	0.594
ETV	PLA	-1.89	-5.56 - 0.72	-0.95	-1.68 - -0.21	-1.01	-1.69 - -0.3	0.516
ETV	TDF	0.46	-0.25 - 1.2	0.22	-0.6 - 1.07	0.37	-0.15 - 0.9	0.635
LAM	LDT	0.34	-0.01 - 0.72	0.46	-0.81 - 1.77	0.34	0.01 - 0.69	0.864
LAM	PEG	0.64	0.1 - 1.21	1.26	0.23 - 2.3	0.77	0.31 - 1.27	0.284
LAM	PLA	-0.81	-1.76 - 0.09	-1.21	-2.18 - -0.26	-1.01	-1.65 - -0.34	0.55
PEG	TDF	-0.69	-1.5 - 0.08	-0.08	-0.94 - 0.79	-0.39	-0.99 - 0.16	0.297
HBeAg sero-conversion								
ADV	ETV	-0.46	-1.88 - 0.88	0.03	-0.55 - 0.6	-0.04	-0.56 - 0.48	0.509
ADV	LAM	-0.64	-1.69 - 0.4	0.15	-0.41 - 0.72	-0.03	-0.52 - 0.48	0.194
ADV	LDT	0.52	-0.58 - 1.65	0.25	-0.41 - 0.91	0.31	-0.23 - 0.89	0.684
ADV	PLA	-0.84	-1.76 - 0.06	-1.29	-2.28 - -0.36	-1.02	-1.7 - -0.39	0.484
ADV	TDF	0.39	-0.22 - 1.02	-0.02	-0.85 - 0.83	0.25	-0.23 - 0.73	0.435
ETV	LAM	-0.01	-0.39 - 0.41	0.12	-0.71 - 0.9	0.01	-0.33 - 0.37	0.784
ETV	PLA	-1.89	-5.39 - 0.8	-0.95	-1.68 - -0.24	-0.99	-1.7 - -0.32	0.501
ETV	TDF	0.29	-0.39 - 1	0.27	-0.43 - 1.01	0.28	-0.19 - 0.78	0.947
LAM	LDT	0.32	-0.03 - 0.72	0.6	-0.62 - 1.83	0.34	0.02 - 0.72	0.685
LAM	PEG	0.69	0.14 - 1.25	1.22	0.21 - 2.23	0.8	0.35 - 1.3	0.346
LAM	PLA	-1.13	-2.05 - -0.34	-0.88	-1.89 - 0.1	-0.99	-1.65 - -0.39	0.685
PEG	TDF	-0.8	-1.67 - 0.03	-0.28	-1.08 - 0.51	-0.53	-1.1 - 0.01	0.35

Abbreviations: Virologic response, undetectable HBV DNA level; ALT norm, normalization of serum alanine aminotransferase; HBeAg sero, hepatitis B e antigen seroconversion; HBeAg loss, hepatitis B e antigen loss; HBsAg loss, hepatitis B surface antigen loss; OR : Odds Ratio, CI : Credible Interval ADV, adefovir; ETV, entecavir; LAM, lamivudine; PEG, pegylated interferon-alfa; PLA, placebo; TAF, tenofovir alafenamide; TBV, telbivudine; TDF, tenofovir disoproxil fumarate; ADVTBV, ETVPEG, ETVPEG1, ETVTDF, LAMADV, LAMADV1, LAMADV2, LAMTBV, LAMPEG, LAMPEG1, LAMPEG2, PEGADV, PEGADV1, PEGADV2, PEGADV3, PEGETV, TDFPEG1 and TDFPEG2 code for different combinations of antiviral agents with full details in Appendix C.
