# Supplementary material for: Antiviral treatment for treatment-naïve chronic hepatitis B: systematic review and network meta-analysis of randomized controlled trials
Source: Syst Rev. 2019 Aug 19;8:207. doi: 10.1186/s13643-019-1126-1 (PMC6699129; doi:10.1186/s13643-019-1126-1)
Supplement: Supplementary file 6 — Relative effects on outcomes of all pairs of interventions in Odds Ratios (95% credible intervals) as calculated from the network meta-analyses using fixed effects models. (PDF 122 kb) [file 13643_2019_1126_MOESM6_ESM.pdf]

Appendix F: Relative effects on outcomes of all pairs of interventions in Odds Ratios (95% credible intervals) as calculated from the network meta-analyses using fixed effects models

**Virologic Response**: HBeAg-positive population in the left lower triangle & HBeAg-negative population in the right upper triangle												
**ADV**			1.83 (0.06, 20.24)	1.53 (0.04, 23.82)	0.38 (0.01, 4)				1.34 (0.04, 14.84)	0.01 (0, 0.06)	11.01 (4.15, 28.96)	9.25 (5.61, 15.76)
0.43 (0.26, 0.68)	**ADV2**											
0.39 (0.18, 0.86)	0.92 (0.36, 2.31)	**ADVTBV**										
0.15 (0.09, 0.25)	0.36 (0.18, 0.71)	0.39 (0.17, 0.87)	**ETV**	0.85 (0.23, 3.09)	0.21 (0.14, 0.3)				0.73 (0.41, 1.3)	0.01 (0, 0.02)	6.1 (0.45, 211.59)	5.1 (0.43, 172.22)
0.08 (0.04, 0.18)	0.2 (0.08, 0.49)	0.22 (0.08, 0.59)	0.56 (0.31, 0.99)	**ETVTDF**	0.24 (0.06, 0.96)				0.86 (0.21, 3.68)	0.01 (0, 0.04)	7.32 (0.39, 307.99)	6.11 (0.38, 244.89)
0.43 (0.26, 0.74)	1.02 (0.5, 2.08)	1.12 (0.5, 2.48)	2.87 (2.28, 3.61)	5.12 (2.76, 9.57)	**LAM**				3.55 (2.34, 5.41)	0.03 (0.01, 0.09)	29.52 (2.29, 1013.11)	24.54 (2.19, 818.3)
0.2 (0.1, 0.4)	0.47 (0.2, 1.11)	0.51 (0.21, 1.32)	1.33 (0.79, 2.21)	2.36 (1.09, 5.13)	0.46 (0.29, 0.73)	**LAMADV1**						
0.25 (0.12, 0.51)	0.58 (0.24, 1.39)	0.63 (0.24, 1.64)	1.63 (0.92, 2.82)	2.89 (1.3, 6.46)	0.57 (0.34, 0.93)	1.23 (0.74, 2.03)	**LAMADV2**					
0.3 (0.11, 0.8)	0.71 (0.24, 2.08)	0.77 (0.25, 2.41)	1.99 (0.85, 4.66)	3.55 (1.26, 9.88)	0.69 (0.31, 1.59)	1.5 (0.59, 3.89)	1.23 (0.47, 3.22)	**LAMTBV**				
0.21 (0.12, 0.36)	0.49 (0.24, 1.01)	0.54 (0.24, 1.18)	1.38 (1, 1.88)	2.45 (1.28, 4.73)	0.48 (0.38, 0.6)	1.04 (0.63, 1.73)	0.85 (0.49, 1.48)	0.69 (0.31, 1.54)	**TBV**	0.01 (0, 0.03)	8.34 (0.62, 296.8)	6.98 (0.6, 235.54)
66.06 (11.69, 1836.09)	155.05 (27.71, 4296.41)	172.34 (24.81, 5073.69)	438.04 (70.47, 12724.24)	801.05 (113.8, 22781.85)	153.64 (24.45, 4417.57)	335.63 (49.87, 9966.97)	271.94 (40.04, 7927.08)	225.07 (29.73, 6855.64)	319.93 (50.92, 9248.77)	**PLA**	1058.56 (138.32, 32609.98)	874.13 (140.77, 26039.89)
0.08 (0.05, 0.13)	0.19 (0.09, 0.38)	0.2 (0.08, 0.5)	0.52 (0.31, 0.9)	0.94 (0.43, 2.07)	0.18 (0.1, 0.32)	0.39 (0.19, 0.83)	0.32 (0.15, 0.7)	0.26 (0.1, 0.71)	0.38 (0.21, 0.69)	0 (0, 0.01)	**TAF**	0.84 (0.38, 1.94)
0.07 (0.05, 0.11)	0.16 (0.09, 0.31)	0.18 (0.08, 0.42)	0.46 (0.29, 0.73)	0.82 (0.4, 1.7)	0.16 (0.1, 0.26)	0.35 (0.18, 0.69)	0.28 (0.14, 0.58)	0.23 (0.09, 0.6)	0.33 (0.2, 0.56)	0 (0, 0.01)	0.88 (0.65, 1.18)	**TDF**
**ALT Normalization**: HBeAg-positive population in the left lower triangle & HBeAg-negative population in the right upper triangle												
**ADV**			3.52 (1.07, 12.41)	3.01 (0.64, 14.38)	2.29 (0.72, 7.7)				3.6 (1.08, 12.99)	0.15 (0.07, 0.3)	2.19 (1.24, 3.9)	1.03 (0.71, 1.5)

0.79 (0.52, 1.21)	**ADV2**											
0.66 (0.22, 1.75)	0.83 (0.26, 2.38)	**ADVTBV**										
0.53 (0.33, 0.85)	0.67 (0.37, 1.23)	0.81 (0.29, 2.44)	**ETV**	0.85 (0.32, 2.18)	0.65 (0.46, 0.91)				1.02 (0.6, 1.75)	0.04 (0.01, 0.11)	0.62 (0.16, 2.32)	0.29 (0.08, 1.03)
1.33 (0.64, 2.79)	1.68 (0.74, 3.88)	2.06 (0.64, 7.06)	2.49 (1.42, 4.48)	**ETVTDF**	0.77 (0.28, 2.14)				1.2 (0.41, 3.63)	0.05 (0.01, 0.2)	0.74 (0.14, 3.85)	0.34 (0.07, 1.71)
0.89 (0.55, 1.45)	1.13 (0.62, 2.05)	1.36 (0.5, 4.05)	1.67 (1.3, 2.16)	0.67 (0.35, 1.24)	**LAM**				1.57 (1.05, 2.38)	0.07 (0.02, 0.16)	0.95 (0.25, 3.44)	0.45 (0.13, 1.52)
0.39 (0.2, 0.76)	0.49 (0.23, 1.06)	0.6 (0.19, 1.98)	0.73 (0.43, 1.25)	0.29 (0.13, 0.64)	0.44 (0.27, 0.7)	**LAMADV1**						
0.3 (0.14, 0.64)	0.38 (0.16, 0.88)	0.46 (0.14, 1.6)	0.57 (0.3, 1.07)	0.23 (0.09, 0.53)	0.34 (0.19, 0.61)	0.78 (0.41, 1.44)	**LAMADV2**					
0.73 (0.24, 2.15)	0.92 (0.29, 2.86)	1.12 (0.27, 4.79)	1.37 (0.49, 3.76)	0.55 (0.17, 1.73)	0.82 (0.3, 2.17)	1.86 (0.62, 5.6)	2.41 (0.75, 7.57)	**LAMTBV**				
0.6 (0.35, 0.99)	0.75 (0.4, 1.4)	0.91 (0.33, 2.73)	1.12 (0.8, 1.57)	0.45 (0.23, 0.86)	0.67 (0.53, 0.84)	1.53 (0.91, 2.59)	1.97 (1.05, 3.77)	0.82 (0.31, 2.21)	**TBV**	0.04 (0.01, 0.11)	0.61 (0.15, 2.32)	0.29 (0.08, 1.02)
5.61 (3.57, 9.08)	7.08 (4.39, 11.93)	8.61 (2.94, 27.22)	10.53 (6.08, 18.52)	4.21 (1.88, 9.39)	6.29 (3.69, 10.98)	14.43 (7.02, 29.99)	18.65 (8.3, 41.97)	7.71 (2.55, 23.82)	9.42 (5.33, 17.08)	**PLA**	14.58 (5.94, 36.84)	6.84 (3.15, 15.58)
0.47 (0.29, 0.78)	0.6 (0.31, 1.12)	0.72 (0.24, 2.3)	0.89 (0.53, 1.48)	0.35 (0.16, 0.76)	0.53 (0.31, 0.91)	1.21 (0.59, 2.49)	1.56 (0.7, 3.52)	0.64 (0.21, 2)	0.79 (0.44, 1.42)	0.08 (0.04, 0.16)	**TAF**	0.47 (0.31, 0.72)
0.68 (0.45, 1.02)	0.86 (0.48, 1.52)	1.03 (0.37, 3.19)	1.27 (0.83, 1.96)	0.51 (0.25, 1.03)	0.76 (0.48, 1.22)	1.74 (0.9, 3.39)	2.25 (1.06, 4.8)	0.93 (0.32, 2.8)	1.14 (0.69, 1.9)	0.12 (0.07, 0.21)	1.44 (1.08, 1.93)	**TDF**

**HBeAg seroconversion** in the left lower triangle & **HBeAg loss** in the right upper triangle													
**ADV**	1.17 (0.72, 1.9)	1.19 (0.48, 2.86)	1.05 (0.6, 1.81)	0.69 (0.32, 1.47)	0.99 (0.58, 1.71)	0.99 (0.47, 2.1)	0.96 (0.42, 2.1)	0.54 (0.17, 1.62)	1.38 (0.79, 2.44)	0.38 (0.22, 0.64)	2.06 (0.99, 4.35)	1.74 (0.96, 3.21)	
0.88 (0.47, 1.63)	**ADV2**	1.03 (0.37, 2.73)	0.89 (0.45, 1.79)	0.59 (0.25, 1.38)	0.85 (0.43, 1.69)	0.85 (0.36, 1.99)	0.82 (0.33, 2)	0.46 (0.13, 1.49)	1.18 (0.59, 2.38)	0.33 (0.18, 0.57)	1.77 (0.75, 4.21)	1.5 (0.71, 3.14)	
0.88 (0.36, 2.19)	1 (0.35, 2.94)	**ADVTBV**	0.88 (0.36, 2.21)	0.58 (0.21, 1.67)	0.83 (0.35, 2.05)	0.83 (0.3, 2.36)	0.8 (0.28, 2.34)	0.45 (0.12, 1.65)	1.16 (0.49, 2.83)	0.32 (0.12, 0.87)	1.74 (0.59, 5.19)	1.46 (0.55, 4.03)	
1.07 (0.67, 1.72)	1.21 (0.58, 2.55)	1.21 (0.47, 3.01)	**ETV**	0.66 (0.4, 1.1)	0.95 (0.72, 1.25)	0.95 (0.53, 1.69)	0.92 (0.47, 1.73)	0.52 (0.17, 1.41)	1.32 (0.93, 1.89)	0.37 (0.19, 0.67)	1.98 (1.01, 3.9)	1.66 (0.99, 2.81)	

1.88 (0.9, 3.92)	2.13 (0.84, 5.43)	2.14 (0.72, 6.19)	1.75 (1.02, 3.06)	**ETVTDF**	1.43 (0.8, 2.56)	1.43 (0.66, 3.1)	1.38 (0.6, 3.12)	0.77 (0.24, 2.42)	1.99 (1.08, 3.75)	0.55 (0.25, 1.23)	2.99 (1.28, 7.01)	2.52 (1.22, 5.23)	
1.11 (0.7, 1.78)	1.26 (0.61, 2.62)	1.26 (0.5, 3.07)	1.04 (0.79, 1.37)	0.59 (0.32, 1.1)	**LAM**	1 (0.6, 1.66)	0.97 (0.53, 1.72)	0.54 (0.19, 1.42)	1.4 (1.11, 1.77)	0.39 (0.21, 0.7)	2.09 (1.03, 4.25)	1.76 (0.99, 3.1)	
0.37 (0.17, 0.81)	0.42 (0.15, 1.12)	0.42 (0.13, 1.31)	0.35 (0.15, 0.77)	0.2 (0.07, 0.53)	0.34 (0.15, 0.72)	**LAMADV**							
1.31 (0.63, 2.76)	1.5 (0.59, 3.78)	1.49 (0.51, 4.26)	1.23 (0.65, 2.33)	0.7 (0.3, 1.63)	1.18 (0.67, 2.11)	3.53 (1.35, 9.46)	**LAMADV1**	0.96 (0.53, 1.73)	0.54 (0.17, 1.61)	1.4 (0.8, 2.44)	0.39 (0.18, 0.84)	2.09 (0.87, 5.03)	1.76 (0.82, 3.78)
1.48 (0.66, 3.44)	1.69 (0.63, 4.64)	1.69 (0.54, 5.14)	1.39 (0.67, 2.94)	0.79 (0.31, 2.02)	1.34 (0.68, 2.69)	4 (1.44, 11.62)	1.13 (0.57, 2.3)	**LAMADV2**	0.56 (0.17, 1.75)	1.45 (0.78, 2.74)	0.4 (0.17, 0.93)	2.16 (0.86, 5.47)	1.82 (0.82, 4.18)
2.14 (0.71, 7.17)	2.45 (0.69, 9.31)	2.43 (0.64, 10.03)	2 (0.7, 6.46)	1.14 (0.35, 4.14)	1.92 (0.71, 6.01)	5.75 (1.62, 22.87)	1.63 (0.51, 5.74)	1.44 (0.42, 5.36)	**LAMTBV**	2.57 (1, 7.27)	0.72 (0.23, 2.35)	3.87 (1.18, 13.66)	3.25 (1.07, 10.66)
0.8 (0.48, 1.33)	0.91 (0.43, 1.94)	0.91 (0.37, 2.21)	0.75 (0.52, 1.09)	0.43 (0.22, 0.83)	0.72 (0.57, 0.93)	2.16 (0.97, 4.92)	0.61 (0.33, 1.14)	0.54 (0.26, 1.1)	0.38 (0.12, 1.01)	**TBV**	0.28 (0.15, 0.52)	1.49 (0.72, 3.12)	1.26 (0.69, 2.31)
2.9 (1.58, 5.49)	3.3 (1.6, 7.08)	3.29 (1.16, 9.31)	2.72 (1.47, 5.21)	1.55 (0.68, 3.68)	2.61 (1.47, 4.85)	7.83 (3.09, 20.6)	2.22 (0.98, 5.13)	1.96 (0.79, 4.88)	1.36 (0.38, 4.43)	3.61 (1.95, 7.01)	**PLA**	5.42 (2.33, 12.56)	4.56 (2.22, 9.49)
0.55 (0.28, 1.09)	0.63 (0.25, 1.55)	0.63 (0.21, 1.84)	0.52 (0.26, 1.04)	0.3 (0.12, 0.72)	0.5 (0.24, 1.01)	1.49 (0.55, 4.12)	0.42 (0.17, 1.06)	0.37 (0.14, 0.99)	0.26 (0.07, 0.89)	0.69 (0.32, 1.46)	0.19 (0.08, 0.45)	**TAF**	0.84 (0.54, 1.29)
0.73 (0.46, 1.15)	0.83 (0.39, 1.74)	0.83 (0.31, 2.15)	0.68 (0.42, 1.1)	0.39 (0.19, 0.81)	0.66 (0.39, 1.1)	1.97 (0.83, 4.75)	0.56 (0.26, 1.2)	0.49 (0.21, 1.15)	0.34 (0.1, 1.06)	0.91 (0.52, 1.58)	0.25 (0.12, 0.5)	1.31 (0.8, 2.21)	**TDF**

HBsAg loss for HBeAg-positive population

**ADV**	2.9 (0.05, 271.54)	3.85 (0.03, 640.68)	1.85 (0.01, 329.3)	1.69 (0, 1130.85)	1.8 (0, 1261.02)	1.8 (0.01, 494.34)	0.66 (0, 332.63)	13.33 (0.19, 2314.97)	4.41 (0.25, 178.38)
0.34 (0, 22.06)	**ETV**	1.31 (0.11, 15.76)	0.63 (0.05, 7.79)	0.58 (0.01, 63.34)	0.59 (0, 65.36)	0.61 (0.02, 15.05)	0.23 (0, 15.45)	4.53 (0.07, 557.31)	1.58 (0.09, 30.77)

0.26 (0, 32.13)	0.76 (0.06, 9.37)	**ETVTDF**	0.48 (0.01, 17.23)	0.43 (0, 89.87)	0.45 (0, 97.49)	0.47 (0.01, 26.73)	0.17 (0, 20.11)	3.47 (0.03, 718.78)	1.18 (0.03, 56.56)
0.54 (0, 77.74)	1.59 (0.13, 20.71)	2.1 (0.06, 75.21)	**LAM**	0.93 (0.02, 49.96)	0.94 (0.02, 52.48)	0.98 (0.13, 7.42)	0.38 (0.01, 9.99)	7.24 (0.06, 1694.37)	2.53 (0.05, 129.33)
0.59 (0, 325.27)	1.73 (0.02, 198.06)	2.3 (0.01, 483.26)	1.08 (0.02, 61.42)	**LAMADV1**	1.03 (0.02, 61.82)	1.05 (0.01, 98.11)	0.39 (0, 76.6)	8.2 (0.01, 6590.4)	2.86 (0.01, 665.43)
0.56 (0, 323.9)	1.7 (0.02, 206.83)	2.23 (0.01, 498.84)	1.07 (0.02, 62.58)	0.97 (0.02, 61.37)	**LAMADV2**	1.01 (0.01, 94.99)	0.39 (0, 76.92)	7.85 (0.01, 6778.66)	2.7 (0.01, 693.81)
0.55 (0, 114.71)	1.64 (0.07, 44.14)	2.12 (0.04, 136.67)	1.02 (0.13, 7.72)	0.96 (0.01, 88.89)	0.99 (0.01, 84.19)	**TBV**	0.38 (0, 17.85)	7.4 (0.04, 2548.22)	2.61 (0.03, 211.32)
1.5 (0, 821.17)	4.35 (0.06, 468.91)	5.75 (0.05, 1160.17)	2.67 (0.1, 134.84)	2.53 (0.01, 655.63)	2.57 (0.01, 747.77)	2.64 (0.06, 208.83)	**PLA**	20.47 (0.05, 15819.77)	6.86 (0.04, 1758.07)
0.08 (0, 5.39)	0.22 (0, 15.21)	0.29 (0, 39.2)	0.14 (0, 18.17)	0.12 (0, 67.34)	0.13 (0, 67.65)	0.14 (0, 27.08)	0.05 (0, 18.71)	**TAF**	0.37 (0.01, 7.23)
0.23 (0.01, 4.01)	0.63 (0.03, 11.07)	0.84 (0.02, 35.92)	0.4 (0.01, 18.37)	0.35 (0, 89.03)	0.37 (0, 87.88)	0.38 (0, 29.05)	0.15 (0, 23.22)	2.67 (0.14, 127.9)	**TDF**

Footnotes: Treatments are reported in alphabetical order.
Empty cells in the right upper triangles are due to lack of studies comparing the treatment pairs in the virologic response and ALT normalization networks of the HBeAg-negative population; and the lack of studies comparing the treatment pairs in the HBeAg loss network.
Data are reported as an odds ratio (95% CI). Odd ratios higher than 1 favor the column-defining treatment.
Significant results are in red.

Abbreviations: HBeAg seroconversion, hepatitis B e antigen seroconversion; HBeAg loss, hepatitis B e antigen loss; HBsAg loss, hepatitis B surface antigen loss; ADV, adefovir 10mg daily; ADV2, adefovir 30mg daily; ETV, entecavir; LAM, lamivudine; TBV, telbivudine; PLA, placebo; TAF, tenofovir alafenamide; TDF, tenofovir disoproxil fumarate; ADVTBV, ETVTDF, LAMADV, LAMADV1, LAMADV2, and LAMTBV code for different combinations of antiviral agents with full details in Appendix C.
